# Recurrent hotspot mutations in *HRAS* Q61 and PI3K-AKT pathway genes as drivers of breast adenomyoepitheliomas

**DOI:** 10.1038/s41467-018-04128-5

**Published:** 2018-05-08

**Authors:** Felipe C. Geyer, Anqi Li, Anastasios D. Papanastasiou, Alison Smith, Pier Selenica, Kathleen A. Burke, Marcia Edelweiss, Huei-Chi Wen, Salvatore Piscuoglio, Anne M. Schultheis, Luciano G. Martelotto, Fresia Pareja, Rahul Kumar, Alissa Brandes, Dan Fan, Thais Basili, Arnaud Da Cruz Paula, John R. Lozada, Pedro Blecua, Simone Muenst, Achim A. Jungbluth, Maria P. Foschini, Hannah Y. Wen, Edi Brogi, Juan Palazzo, Brian P. Rubin, Charlotte K. Y. Ng, Larry Norton, Zsuzsanna Varga, Ian O. Ellis, Emad A. Rakha, Sarat Chandarlapaty, Britta Weigelt, Jorge S. Reis-Filho

**Affiliations:** 1 Department of Pathology, Memorial Sloan Kettering Cancer Center, New York, NY 10065 USA; 2 Hospital Israelita Albert Einstein, Instituto Israelita de Ensino e Pesquisa, São Paulo, 05652-900 Brazil; 3 Instituto do Cancer do Estado de São Paulo, São Paulo, 01246-000 Brazil; 4 Department of Pathology, Fudan University Shanghai Cancer Center, 200032 Shanghai, PR China; 5 Department of Pathology, Patras General Hospital, 263 32 Patras, Greece; 6 Human Oncology and Pathogenesis Program, Memorial Sloan Kettering Cancer Center, New York, NY 10065 USA; 7 Institute of Pathology and Medical Genetics, University Hospital Basel, 4031 Basel, Switzerland; 8 Department of Oncology, Xiangya Hospital, Central South University, 410008 Changsha, Hunan Province PR China; 9 Department of Radiation Oncology, Memorial Sloan Kettering Cancer Center, New York, NY 10065 USA; 10 Department of Biomedical and Neuromotor Sciences, University of Bologna, Section of Bellaria Hospital, 40139 Bologna, Italy; 11 Department of Pathology, Thomas Jefferson University Hospital, Philadelphia, PA 19107 USA; 12 Department of Pathology, Robert J. Tomsich Pathology and Laboratory Medicine Institute, Cleveland Clinic, Cleveland, OH 44195 USA; 13 Department of Biomedicine, University of Basel, 4001 Basel, Switzerland; 14 Department of Medicine, Memorial Sloan Kettering Cancer Center, New York, NY 10065 USA; 15 Institute of Surgical Pathology, University Hospital Zurich, 8091 Zurich, Switzerland; 16 Department of Pathology, University of Nottingham, Nottingham, NG7 2RD UK

## Abstract

Adenomyoepithelioma of the breast is a rare tumor characterized by epithelial−myoepithelial differentiation, whose genetic underpinning is largely unknown. Here we show through whole-exome and targeted massively parallel sequencing analysis that whilst estrogen receptor (ER)-positive adenomyoepitheliomas display *PIK3CA* or *AKT1* activating mutations, ER-negative adenomyoepitheliomas harbor highly recurrent codon Q61 *HRAS* hotspot mutations, which co-occur with *PIK3CA* or *PIK3R1* mutations. In two- and three-dimensional cell culture models, forced expression of HRAS^Q61R^ in non-malignant ER-negative breast epithelial cells with or without a *PIK3CA*^H1047R^ somatic knock-in results in transformation and the acquisition of the cardinal features of adenomyoepitheliomas, including the expression of myoepithelial markers, a reduction in E-cadherin expression, and an increase in AKT signaling. Our results demonstrate that adenomyoepitheliomas are genetically heterogeneous, and qualify mutations in *HRAS*, a gene whose mutations are vanishingly rare in common-type breast cancers, as likely drivers of ER-negative adenomyoepitheliomas.

## Abstract (web summary)

Adenomyoepithelioma is a rare tumor of the breast with an unknown genetic basis. Here the authors perform a genomic analysis of adenomyoepitheliomas revealing that their repertoire of somatic mutations vary according to the estrogen receptor (ER) status, and that ER-negative tumors harbor recurrent mutations in *HRAS* and PI3K pathway genes.

## Introduction

Adenomyoepithelioma of the breast is a rare biphasic tumor composed of epithelial and myoepithelial cells^1^, which typically displays a benign clinical course, but may recur locally^2^ and/or metastasize^3^. Phenotypically, adenomyoepitheliomas are heterogeneous. The epithelial component may express estrogen receptor (ER) and progesterone receptor (PR); however, a subset of adenomyoepitheliomas lacks the expression of hormone receptors altogether^1^. Both the epithelial and myoepithelial compartments can expand and undergo malignant transformation, histologically characterized by nuclear atypia, mitotic activity, and/or necrosis^1,2,4^. Importantly, however, metastases have been documented even in cases lacking a histologically overt malignant component^3^. Interestingly, most invasive breast cancers arising in adenomyoepitheliomas display a triple-negative phenotype (ER-, PR- and HER2-negative) and metaplastic features^1^.

The genomic landscape of breast cancers has been extensively investigated (reviewed in Ng et al.^5^). Large-scale massively parallel sequencing studies have revealed that breast cancers display a complex repertoire of somatic mutations, that *TP53* (37%), *PIK3CA* (36%), and *GATA3* (11%) are the only three genes recurrently mutated in >10% of unselected breast cancers, and that the repertoire of somatic mutations differs between ER-positive and ER-negative disease; however, no pathognomonic mutations underpinning ER-positive or ER-negative breast cancers have been identified^6,7^. These analyses, however, primarily focused on the common forms of breast cancer^6–8^, whereas the genetic characteristics of rare forms of breast tumors, including those with myoepithelial differentiation, remain largely unexplored^5^. In fact, genomic analyses of adenomyoepitheliomas, based on case reports or small series of cases, have demonstrated the presence of a t(8;16)(p23;q21) chromosomal translocation in a single case^9^ or a *TP53* R270C missense mutation in another^10^. The landscape of somatic genetic alterations of adenomyoepitheliomas, however, has yet to be characterized.

Here we report on a combination of whole-exome (WES) and targeted capture massively parallel sequencing analyses that revealed that adenomyoepitheliomas are genetically heterogeneous and that, akin to common-type breast cancers, their repertoire of somatic mutations vary according to their ER status. In ER-negative adenomyoepitheliomas, recurrent *HRAS* Q61 hotspot mutations co-occur with mutations affecting PI3K pathway genes. In non-malignant triple-negative breast epithelial cells with or without a somatic knock-in of a *PIK3CA* H1047R mutation, forced expression of mutant HRAS promotes growth advantage, the acquisition of features consistent with myoepithelial differentiation, and activation of PI3K-AKT and MAPK signaling pathways, likely acting as a driver of ER-negative adenomyoepitheliomas.

## Results

### Clinical and histologic features of adenomyoepitheliomas

**Fig. 1:**
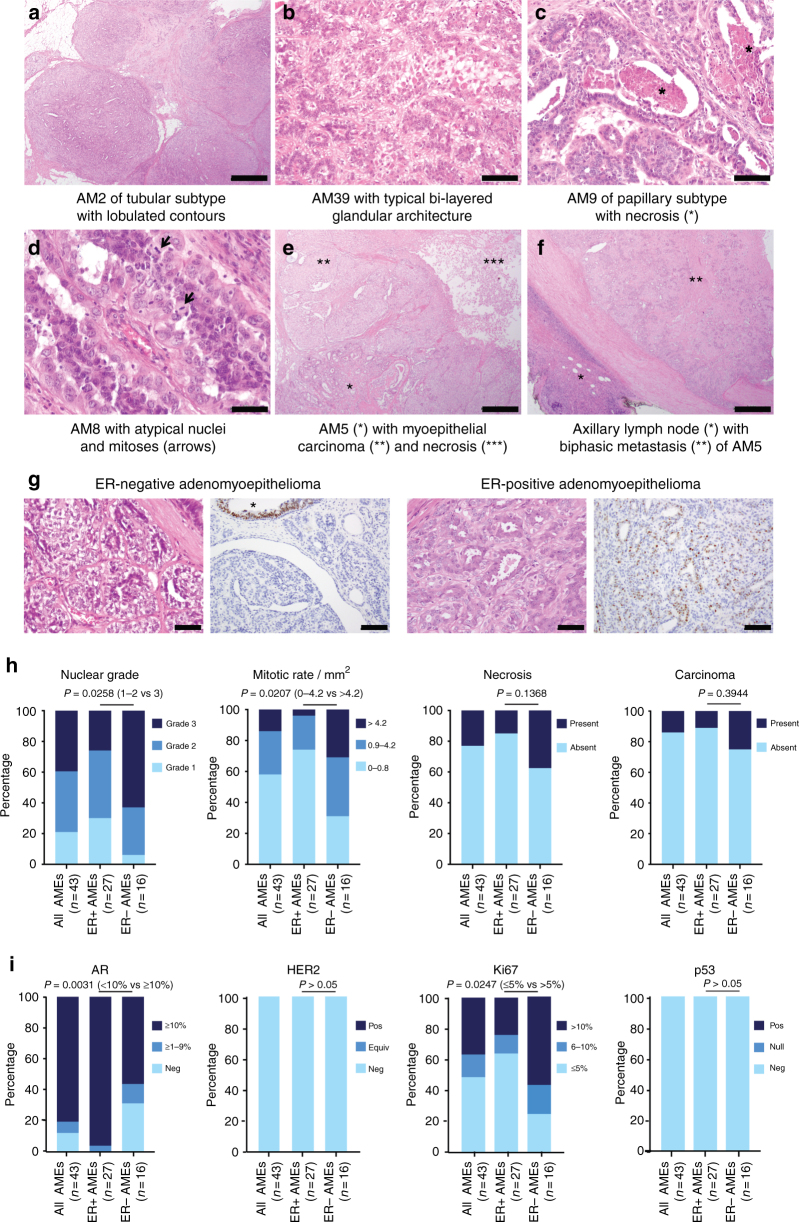
Histologic and immunohistochemical features of adenomyoepitheliomas. **a−f** Representative micrographs of hematoxylin-and-eosin (H&E)-stained adenomyoepitheliomas included in this study. **a** Low-power magnification of AM2, a multilobulated lesion, of tubular architectural pattern, with well-circumscribed borders (scale bar, 1 mm). **b** Intermediate-power magnification of AM39 displaying the typical bi-layered glandular architecture of adenomyoepitheliomas, comprising abluminal myoepithelial cells with clear cytoplasm and inner cuboidal epithelial cells with eosinophilic cytoplasm and apical snouts (scale bar, 200 μm). **c** AM9 displaying areas of comedo-like necrosis (*, scale bar, 200 μm). **d** AM8 displaying nuclear atypia and mitotic figures (arrowheads, scale bar, 100 μm). **e** AM5 displaying an adenomyoepithelioma component (lower left corner, *) in association with a larger high-grade myoepithelial carcinoma (**), with large central necrosis in the upper right corner (***, scale bar, 1 mm). **f** Axillary lymph node metastasis of AM5 (scale bar, 1 mm), where the biphasic architecture is maintained. *, residual lymph node; **, metastatic lesion. **g** Representative micrographs of estrogen receptor (ER)-negative and ER-positive adenomyoepitheliomas. On the left, H&E stain of each case (scale bars, 100 μm). On the right, ER immunohistochemistry results. Note the internal positive control (*) in the ER-negative case. **h** Stacked bar plots showing the frequency of histologic features indicative of a more aggressive behavior (nuclear grade, mitotic rate, and necrosis) and of the presence of associated carcinoma according to ER status (ER-positive versus ER-negative comparisons were performed using two-tailed Fisher’s exact tests). The histologic features are color-coded according to the legends. AME, adenomyoepithelioma. **i** Stacked bar plots showing the frequency of the expression of androgen receptor, HER2, Ki67, and p53 according to ER status (ER-positive versus ER-negative comparisons were performed using two-tailed Fisher’s exact tests). AME, adenomyoepithelioma; AR, androgen receptor; Equiv, equivocal; Neg, negative; Pos, positive

Adenomyoepitheliomas were retrieved from the authors’ institutions after approval by the local Institutional Review Boards (IRBs). Upon central histologic review, 43 cases were considered bona fide adenomyoepitheliomas, of which 18 (42%) displayed atypical histologic features suggestive of a more aggressive behavior (i.e. marked nuclear pleomorphism, high mitotic rate, and/or necrosis^1^; Fig. 1, Supplementary Data 1). Immunohistochemical analysis revealed that 16 adenomyoepitheliomas (37%) lacked ER expression (Fig. 1g, Supplementary Figs. 1a−l), a feature that was significantly associated with nuclear pleomorphism, increased mitotic rate, and higher Ki-67 labeling indices (*P* < 0.05, Fisher’s exact tests, Fig. 1h−i, Supplementary Data 1, Supplementary Fig. 1). AR was expressed in ≥10% of tumor cells (the cut-off employed to select patients for potential anti-androgen therapy^11^) in all but one (26/27, 96%) ER-positive adenomyoepitheliomas, but in 9/16 (56%) ER-negative adenomyoepitheliomas (*P* < 0.01, Fisher’s exact test, Fig. 1i, Supplementary Figs. 1c, i). All adenomyoepitheliomas studied lacked HER2 protein overexpression and displayed focal p53 expression suggestive of an unaltered *TP53* status (Fig. 1i, Supplementary Data 1, Supplementary Figs. 1f, l). As expected, the vast majority of adenomyeopitheliomas (88%) displayed strong p63 expression in the myoepithelial component (Supplementary Figs. 1d, j).

Seven adenomyoepitheliomas (16%) were associated with invasive carcinoma: six present in the primary tumor and one in the ipsilateral breast recurrence (Fig. 1e, Supplementary Data 1, Supplementary Figs. 1m−x). The ER status of paired adenomyoepitheliomas and carcinomas was concordant in all but one case; in the ER-positive AM46, the invasive carcinoma of spindle cell metaplastic type lacked ER expression. Three ER-negative adenomyoepitheliomas developed local recurrences and/or had axillary lymph-node metastases (Supplementary Fig. 1, Supplementary Data 1). Consistent with previous reports of biphasic metastases of adenomyoepitheliomas^3^, the metastases observed in these cases retained the epithelial−myoepithelial phenotype (Fig. 1, Supplementary Fig. 1f), suggesting that at least in some cases, the epithelial and myoepithelial cell populations likely share a common cell of origin with dual-lineage potential.

### Adenomyoepitheliomas harbor recurrent *HRAS* Q61 mutations

To define the genomic landscape of adenomyoepitheliomas, DNA samples extracted from 31 tumor-normal pairs of adenomyoepitheliomas (Supplementary Data 1) were subjected to WES (*n* = 10, median depth of coverage of tumor 159× (range 117×–167×) and normal 95× (range 69×–175×) samples) or targeted capture massively parallel sequencing due to limited yields of DNA available (*n* = 21, median depth of coverage of tumor 434× (range 252×–749×) and normal 401× (range 83×–897×) samples), using the Memorial Sloan Kettering-Integrated Mutation Profiling of Actionable Cancer Targets (MSK-IMPACT). This sequencing assay targets all coding regions of 410 key cancer genes and intronic and regulatory regions of selected genes (Supplementary Data 2). In addition, the *TERT* gene promoter region was investigated in all cases by MSK-IMPACT and/or Sanger sequencing.

Adenomyoepitheliomas displayed a low mutation burden, with a median of 17 (range 5−63) somatic mutations as defined by WES, of which 13.5 (range 4–47) were non-synonymous, whereas the MSK-IMPACT assay detected a median of 2 (range 0–7) somatic mutations, of which 2 (range 0–6) were non-synonymous (Supplementary Data 2). Compared to common forms of invasive breast cancers from The Cancer Genome Atlas (TCGA) dataset (median 46, range 10−396)^6^, adenomyoepitheliomas subjected to WES were found to have significantly fewer somatic mutations (*P* < 0.0001, Wilcoxon test). By WES, ER-negative adenomyoepitheliomas displayed a numerically but not statistically significant higher number of somatic mutations than ER-positive adenomyoepitheliomas (22 (range 6–36) versus 14 (range 5–63), *P* > 0.1, Wilcoxon test). In agreement with the immunohistochemistry analysis, *TP53*, the gene most frequently mutated in breast cancer and mutated in up to 80% of ER-negative breast cancers^6^, was not found to be altered in any of the adenomyoepitheliomas analyzed in this study.

**Fig. 2:**
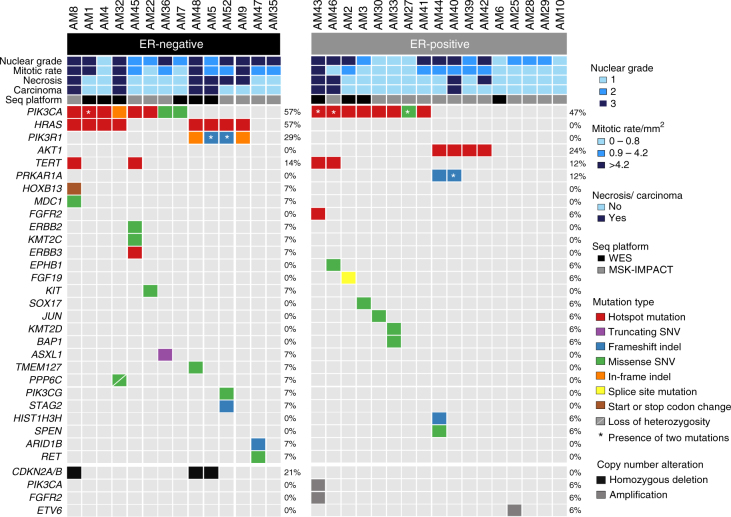
Repertoire of somatic genetic alterations in breast adenomyoepitheliomas. Heatmap depicting the somatic genetic alterations identified in 31 breast adenomyoepitheliomas subjected to whole-exome (WES, *n* = 10) or MSK-IMPACT (*n* = 21) sequencing. Somatic mutations affecting the 410 genes present in the MSK-IMPACT assay are plotted, in decreasing overall mutational frequency, followed by selected genes affected by amplifications or homozygous deletions at the bottom. Cases are shown in columns (estrogen receptor (ER)-negative cases on the left; ER-positive cases on the right), and genes in rows. Histopathologic characteristics and sequencing platforms are shown in the phenotype bars at the top. Genetic alterations are color-coded according to the legend. Loss of heterozygosity (LOH) is represented by a diagonal bar. The presence of two mutations in the same gene is indicated by an asterisk. In cases subjected to WES, hotspot mutations affecting the promoter of *TERT* were investigated by Sanger sequencing. Hotspot mutations were obtained from Chang et al.^57^. Seq platform, sequencing platform employed; SNV, single nucleotide variant; indel, small insertion or deletion

Despite the low mutation burden, mutational analysis revealed recurrently mutated genes, including *PIK3CA* (16/31, 52%), *HRAS* (8/31, 26%), *AKT1* (4/31, 13%), and *PIK3R1* (4/31, 13%). The *PIK3CA* mutations preferentially affected mutation hotspots (six H1047R, five E545K, and one E542K hotspot mutations). All *HRAS* mutations affected the mutation hotspot Q61, and all *AKT1* mutations were the E17K hotspot mutation (Fig. 2, Supplementary Data 3). In four adenomyoepitheliomas (AM1, AM27, AM43, and AM46), dual *PIK3CA* mutations were identified, and in AM5 and AM52, dual *PIK3R1* small deletions were detected. Of the 38 mutations affecting these four genes detected by WES or MSK-IMPACT, 34 (89%) were found to be clonal by ABSOLUTE^12^ (Supplementary Fig. 2a, Supplementary Data 3). Additional cancer genes recurrently mutated in adenomyoepitheliomas included *TERT* (4/31, 13%, all hotspot promoter mutations) and *PRKAR1A* (2/31, 6%, Fig. 2, Supplementary Data 3**)**. Likely pathogenic mutations affecting single tumors included an *ERBB3* D297Y hotspot mutation in a *PIK3CA*-mutant and *HRAS*-wild-type adenomyoepithelioma (Fig. 2, Supplementary Data 3).

Importantly, however, differences were observed in the repertoire of somatic mutations of adenomyoepitheliomas: *AKT1* hotspot mutations were solely found in ER-positive lesions (24% versus 0%, *P* = 0.1074, Fisher’s exact test), *PIK3R1* small deletions were only detected in ER-negative adenomyoepitheliomas (29% versus 0%, *P* = 0.0318, Fisher’s exact test), and *HRAS* Q61 hotspot mutations were restricted to ER-negative adenomyoepitheliomas (57% versus 0%, *P* = 0.0004, Fisher’s exact test) and always co-occurred with *PIK3CA* or *PIK3R1* somatic mutations (Fig. 2, Supplementary Data 3).

**Fig. 3:**
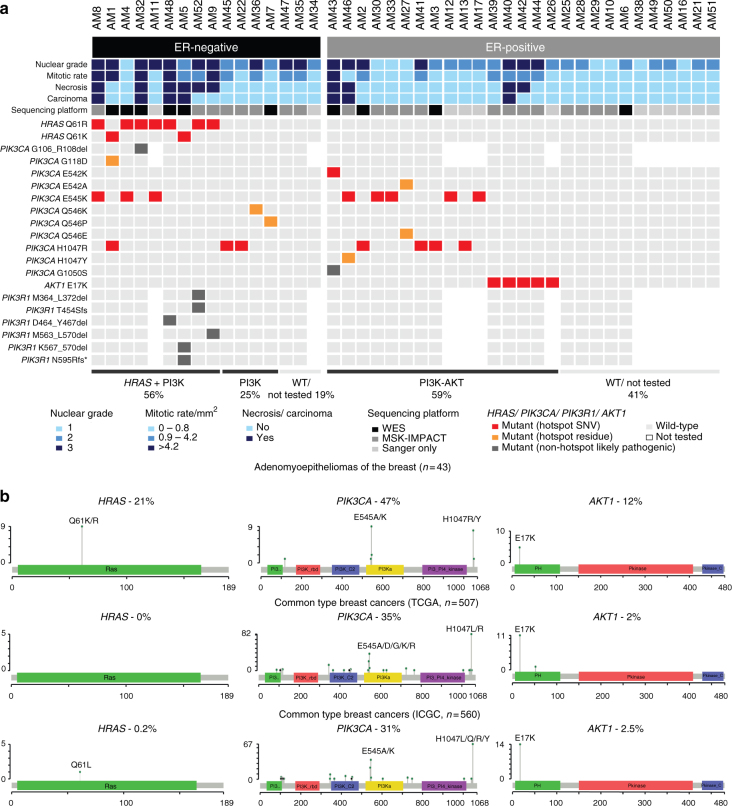
Somatic mutations affecting *HRAS* and PI3K-AKT pathway-related genes in breast adenomyoepitheliomas. **a** Heatmap depicting somatic mutations affecting *HRAS, PIK3CA*, *AKT1,* and *PIK3R1* identified in 43 breast adenomyoepitheliomas by (i) both massively parallel sequencing (WES or MSK-IMPACT) and Sanger sequencing or (ii) Sanger sequencing only. Cases are shown in columns (estrogen receptor (ER)-negative cases on the left; ER-positive cases on the right), and genes in rows. Histopathologic characteristics and sequencing platforms are shown at the top, and color-coded according to the legend at the bottom. Hotspot mutations were obtained from Chang et al.^57^. SNV, single nucleotide variant; WES, whole-exome sequencing; WT, wild-type. **b** Spectrum of somatic mutations affecting *HRAS, PIK3CA*, and *AKT1* identified in the 43 breast adenomyoepitheliomas analyzed in this study, and in unselected breast cancers from The Cancer Genome Atlas (TCGA, *n* = 507)^6^ and International Cancer Genome Consortium (ICGC, *n* = 560)^7^ studies. Diagrams representing the protein domains of HRAS encoded by *HRAS* (left), p110α encoded by *PIK3CA* (middle), and AKT1 encoded by *AKT1* (right). The mutations in these three genes are shown on the *x*-axis, and the height of each “lollipop” indicates the frequency of the mutation (*y*-axis). Missense mutations are depicted as green circles, and small insertions and deletions are shown in black circles. Plots were generated using MutationMapper on cBioPortal (www.cBioPortal.org) and were manually edited

To validate the *HRAS, PIK3CA*, and *AKT1* mutations identified by WES or MSK-IMPACT, Sanger sequencing of the hotspot loci was performed in the initial 31 cases and 12 additional adenomyoepitheliomas, for which sufficient DNA could not be obtained for massively parallel sequencing. All *PIK3CA*, *HRAS*, and *AKT1* hotspot mutations were validated in the index cases, and four, one, and one mutations were detected in *PIK3CA*, *HRAS*, and *AKT1*, respectively, in the 12 additional cases (Fig. 3a, Supplementary Fig. 2b, Supplementary Data 3). Mutations affecting *PIK3CA, AKT1*, and *PIK3R1* were found to be significantly mutually exclusive (*n* = 43, *P* = 0.0004, CoMEt exact test), whereas all *HRAS* mutations significantly co-occurred with *PIK3CA* or *PIK3R1* mutations (*n* = 43, *P *value for co-occurrence = 0.025, *Z *test).

In ER-positive adenomyoepitheliomas, mutations affecting *PIK3CA* or *AKT1* were found in 59% (16/27) of cases, whereas in ER-negative adenomyoepitheliomas, mutations affecting *PIK3CA or PIK3R1* were detected in 81% of the cases tested (Fig. 3a). Within ER-positive adenomyoepitheliomas, the presence of *PIK3CA* or *AKT1* mutations was significantly associated with marked nuclear pleomorphism and moderate-to-high mitotic rate (both 46% versus 0%, *P* = 0.0216, Fisher’s exact tests), whereas among ER-negative adenomyoepitheliomas, *HRAS* mutations were significantly associated with necrosis (67% versus 0%, *P* = 0.0114, Fisher’s exact test) and high mitotic rate (56% versus 0%, *P* = 0.0337, Fisher’s exact test).

In the whole dataset (*n* = 43, Fig. 3), all *HRAS* (Q61R/K), all *AKT1* (E17K), and 16/24 *PIK3CA* mutations detected were known activating hotspot mutations (Fig. 3a). Of the eight non-hotspot *PIK3CA* mutations, six affected hotspot residues and all were predicted to be likely pathogenic (Supplementary Data 3). While *PIK3CA* mutations are common in breast cancer (approximately 35%), mutations in *AKT1* and *HRAS* were found to be significantly less frequent in unselected breast cancers than in the adenomyoepitheliomas studied here (*P* < 0.05, Fisher’s exact tests, Fig. 3b, Supplementary Data 4)^6,7^. In fact, *HRAS* mutations were not found in any of the common-type breast cancers included in the TCGA study (*n* = 507)^6^, and detected only in one of 560 (0.2%) breast cancers from the whole-genome sequencing analysis carried out by the International Cancer Genome Consortium (ICGC)^7^.

Taken together, adenomyoepitheliomas of the breast are characterized by low mutation rates and the lack of *TP53* mutations. Mutations affecting PI3K-AKT pathway-related genes are frequent across ER-positive and ER-negative adenomyoepitheliomas. *HRAS* Q61 hotspot mutations, which are remarkably rare in breast cancers, are restricted to ER-negative adenomyoepitheliomas and associated with atypical histology indicative of a more aggressive phenotype.

### Adenomyoepitheliomas display limited genomic complexity

Genome-wide copy number analysis revealed a diploid/near-diploid genome in the 31 adenomyoepitheliomas analyzed here (Supplementary Fig. 3a), which harbored fewer copy number alterations (CNAs) than common forms of breast cancers from TCGA^6^ (Supplementary Fig. 3b). Among the most frequent CNAs were losses of 6p22 (6/31, 19%), 9p21 (*CDKN2A*, 4/31, 13%), and 4q31 (*INPP4B*, 2/31, 6%) and gains of 12p12.3 (*ETV6*, 5/31, 16%) and 5p15 (*TERT*, 4/31, 13%, Supplementary Fig. 3a). Losses of 9p21, which have been previously linked to an unfavorable phenotype in breast cancer^13^, were only detected in adenomyoepitheliomas lacking ER expression (29% versus 0%, *P* = 0.0318, Fisher’s exact test, Supplementary Fig. 3c), and displaying histologic features associated with an unfavorable clinical behavior (i.e., necrosis and/or high mitotic rate; 36% versus 0%, *P* = 0.0105, Fisher’s exact test). An exploratory, hypothesis-generating genome-wide analysis revealed that ER-negative adenomyoepitheliomas displayed a significantly higher number of CNAs than ER-positive lesions (*P* = 0.0374, Wilcoxon test, Supplementary Fig. 3d), and adenomyoepitheliomas with atypical histologic features suggestive of a more aggressive behavior harbored significantly more CNAs than those without (*P* = 0.0051, Wilcoxon test, Supplementary Fig. 3e). Moreover, gains of chromosomes 7 and 8 were restricted to ER-negative adenomyoepitheliomas; however, these were low level gains. Further studies are warranted to define their biological and clinical significance.

Homozygous deletions and high-level amplifications were rarely found in adenomyoepitheliomas. Of note, recurrent homozygous deletions affecting *CDKN2A* were found in 3/14 (21%) ER-negative adenomyoepitheliomas (Fig. 2, Supplementary Fig. 3f) and, interestingly, these three cases were found to progress to carcinoma (100% versus 9%, *P* = 0.011, Fisher’s exact test). Amplifications of oncogenes (e.g., *FGFR2* and *PIK3CA)* were found in individual cases (Fig. 2, Supplementary Fig. 3f), but none of the adenomyoepitheliomas studied harbored *HER2* gene amplification, consistent with the lack of HER2 protein overexpression by immunohistochemistry (Fig. 1i).

### Progression of adenomyoepitheliomas

To assess whether progression to a malignant phenotype is associated with the acquisition of additional somatic genetic alterations and/or clonal selection, we analyzed separately microdissected components of the primary tumor, locally recurrent tumor, and/or lymph node metastases of three patients with adenomyoepitheliomas (Supplementary Figs. 1m−x).

**Fig. 4:**
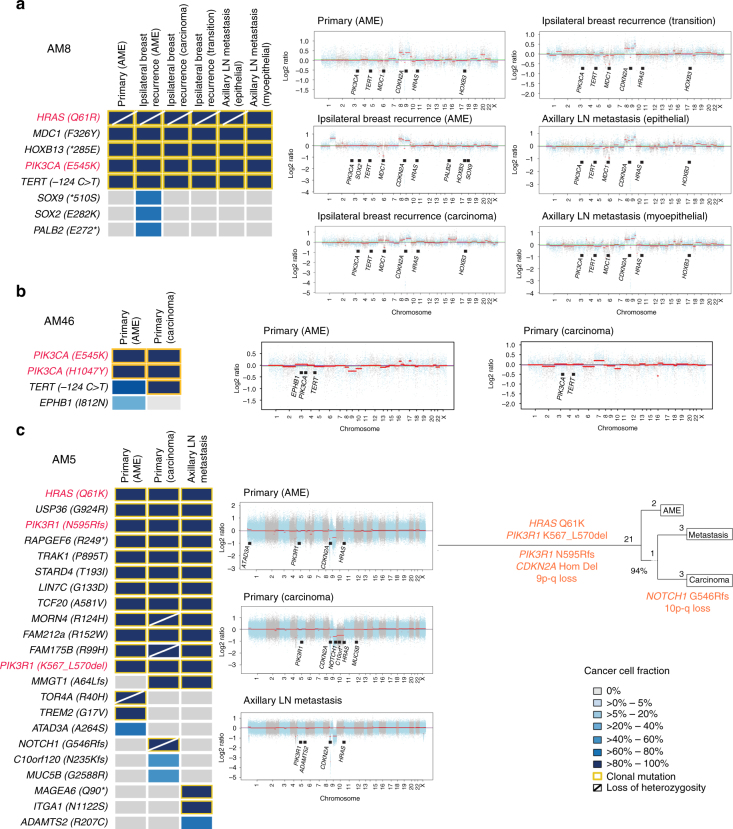
Progression of breast adenomyoepitheliomas. **a−c** On the left, heatmaps depicting the cancer cell fractions of the mutations identified in separately microdissected, histologically distinct components of AM8, AM46, and AM5; and on the right the copy number genome plots of each component. Cancer cell fractions were determined using ABSOLUTE^12^, and color-coded according to the legend. Loss of heterozygosity (LOH) is shown with a diagonal bar. Clonal mutations are highlighted with orange boxes. Mutations affecting *HRAS* and/or PI3K-AKT pathway-related genes are highlighted in red. In copy number genome plots, the genomic position is plotted on the *x*-axis and the Log_2_ ratios on the *y*-axis, and the cancer genes affected by somatic mutations and any gene affected by subclonal somatic mutations are shown according to their genomic position. **a** AM8, where the primary adenomyoepithelioma (AME), separately microdissected components of an ipsilateral relapse in the breast tissue (AME, invasive ductal carcinoma and transition components) and of separately microdissected components of a metachronous axillary lymph node metastasis (epithelial- and myoepithelial-enriched components) were analyzed by MSK-IMPACT sequencing. **b** AM46, where the breast adenomyoepithelioma and spindle cell metaplastic carcinoma components of the primary tumor were analyzed by MSK-IMPACT sequencing. **c** AM5, where the breast adenomyoepithelioma and myoepithelial metaplastic carcinoma components of the primary tumor and a synchronous axillary lymph node metastasis were analyzed by whole-exome sequencing (WES). The phylogenetic tree was constructed using Treeomics^62^. The length of the trunk and branches is proportional to the number of mutations defining each trunk or branches. Likely driver genes and copy number alterations found in the trunk and branches are highlighted in orange. Hom Del, homozygous deletion; LN, lymph node

The separately microdissected components of AM8 and AM46 were subjected to MSK-IMPACT sequencing. In AM8, this analysis revealed clonal *HRAS*, *PIK3CA*, and *TERT* promoter mutations and *CDKN2A* homozygous deletions in all components analyzed, consistent with these being truncal (i.e. present as clonal alterations in all components analyzed) genetic events (Fig. 4a, Supplementary Data 3). In AM46, two clonal *PIK3CA* mutations (Fig. 4b, Supplementary Data 3) were found to be truncal. The adenomyoepithelioma component likely displayed intra-tumor genetic heterogeneity, given that it harbored a subclonal *EPHB1* missense mutation and a subclonal *TERT* promoter hotspot mutation. Interestingly, the latter was found to be clonal in the carcinoma component of this case.

These analyses suggested the presence of intra-tumor genetic heterogeneity within adenomyoepitheliomas and potential clonal selection in the progression to carcinoma. To investigate these hypotheses, we performed WES analysis of the separately microdissected components of AM5. Based on the analysis of the validated somatic genetic alterations, the adenomyoepithelioma and carcinoma components of the primary tumor and the axillary lymph node metastasis shared 11 clonal truncal mutations, including an *HRAS* and two *PIK3R1* mutations, and a *CDKN2A* homozygous deletion (Fig. 4c, Supplementary Data 3), and truncal CNAs including losses of 9p and 9q. Additional private clonal mutations were found in each of the components, including a frameshift mutation affecting *NOTCH1*, which was coupled with loss of heterozygosity of its wild-type allele due to the truncal 9q loss, and a loss of chromosome 10 restricted to the carcinoma. These findings are consistent with the presence of intra-tumor genetic heterogeneity within the components analyzed, and suggest that each component may have undergone branching evolution (Fig. 4c). Importantly, however, the biologic significance of the private genetic alterations identified remains to be defined.

Taken together, our multi-region genetic analyses demonstrate that adenomyoepitheliomas and their respective carcinomatous or metastatic components display remarkable similarities in regards to their mutational and CNA profiles, with a limited number of known driver genetic alterations enriched or solely detected in the carcinomatous or metastatic components of each case. In addition, this analysis is consistent with the notion that *HRAS* Q61 hotspot mutations, *PIK3CA* mutations, *PIK3R1* mutations, and *CDKN2A* homozygous deletions, when present, likely constitute truncal genetic events, whereas *TERT* promoter mutations may constitute early or late events in the development and/or progression of adenomyoepitheliomas.

### Impact of *HRAS*^Q61R^ on non-malignant breast epithelial cells

Given the high prevalence of *HRAS* Q61 and *PIK3CA* mutations in ER-negative adenomyoepitheliomas, and that these mutations co-occur, we sought to define the functional impact of these mutations in non-malignant ER-negative breast epithelial cells. Given that adenomyoepithelioma cell lines or patient-derived xenografts are not commercially available, we investigated the functional impact of forced expression of the *HRAS* Q61R mutation using the non-malignant breast epithelial cells MCF-10A and MCF-12A. We reasoned that these cell lines would constitute an adequate model for ER-negative adenomyoepitheliomas given their triple-negative phenotype, the fact that they are *TP53* wild-type, and MCF-10A cells harbor a *CDKN2A* homozygous deletion^14^, which was the fourth most frequent somatic genetic alteration identified in ER-negative adenomyoepitheliomas (Fig. 2b). In addition to the parental MCF-10A cells (MCF-10A^P^), to define the impact of concurrent *HRAS*^Q61R^ and *PIK3CA* hotspot mutations (i.e., *PIK3CA*^H1047R^ or *PIK3CA*^E545K^), we employed MCF-10A cells where the oncogenic *PIK3CA*^H1047R^ or *PIK3CA*^E545K^ were knocked-in (MCF-10A^H1047R^ and MCF-10A^E545K^, respectively)^15^.

**Fig. 5:**
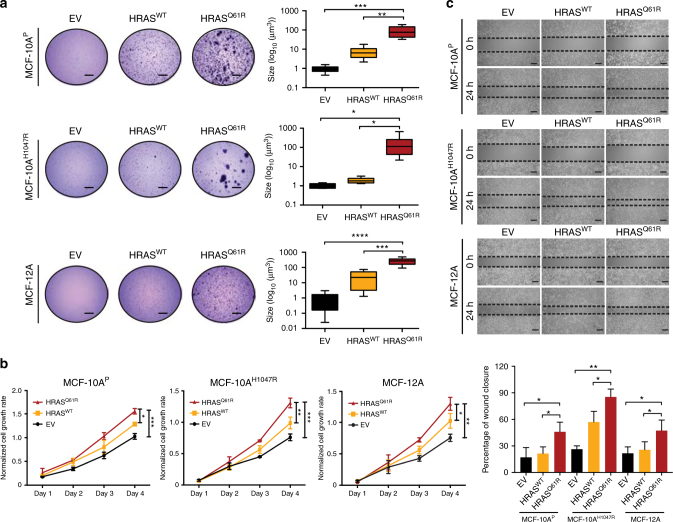
Mutant HRAS^Q61R^ expression induces transformation and growth in non-malignant breast epithelial cells. **a** Representative images of soft agar anchorage-independent growth assay of parental MCF-10A *PIK3CA*-wild-type (MCF-10A^P^), MCF-10A *PIK3CA* H1047R-mutant (MCF-10A^H1047R^), and MCF-12A cells stably expressing empty vector (EV), HRAS-wild-type (HRAS^WT^), or HRAS Q61R-mutant (HRAS^Q61R^) protein (scale bars, 2 mm). Boxplots showing the quantification of the size of colonies (see Methods). The mean value of the size of colonies, and the 75th and 25th percentiles are displayed at the top and bottom of the boxes, respectively. **b** MTT cell proliferation assay of MCF-10A^P^, MCF-10A^H1047R^, and MCF-12A cells stably expressing EV (black), HRAS^WT^ (yellow), or mutant HRAS^Q61R^ (red) protein. **c** The migratory effects of MCF-10A^P^, MCF-10A^H1047R^, and MCF-12A cells stably expressing EV, HRAS^WT^, or mutant HRAS^Q61R^ were analyzed using the wound-healing assay at 0 and 24 h. Scale bars, 500 µm. In **a**−**c**, data are representative of three independent experiments. Error bars, s.d. of mean (*n* = 3). n.s. = not significant, *P < 0.05, **P < 0.01, ***P < 0.001, ****P *<* 0.0001; two-tailed unpaired *t*-tests

As expected, forced expression of mutant HRAS^Q61R^ in monolayer MCF-10A^P^, MCF-10A^H1047R^, and MCF-12A cells resulted in upregulation of GTP-bound HRAS (Supplementary Fig. 4a) and increased ERK1/2 phosphorylation (T202/Y204) as compared to the empty vector (EV) control (Supplementary Fig. 4b). We next investigated the oncogenic impact of *HRAS* Q61R on non-malignant breast epithelial cells. Forced expression of mutant HRAS^Q61R^ in MCF-10A^P^, MCF-10A^H1047R^, and MCF-12A cells resulted in an increase in colony formation and cell proliferation as compared to EV or HRAS^WT^ in soft agar (Fig. 5a) and MTT assays (Fig. 5b), respectively. Moreover, forced expression of mutant HRAS^Q61R^ resulted in a significantly increased migration as compared to EV or HRAS^WT^ in the three cell lines, as demonstrated by a wound-healing assay (Fig. 5c, *P* < 0.05, unpaired *t-*tests). Consistent with the observations made using PIK3CA^H1047R^ cells, forced expression of mutant HRAS in MCF-10A^E545K^ cells also resulted in increased proliferation and migration, as compared to EV and HRAS^WT^ (Supplementary Fig. 5a−c). In addition, mammosphere formation assays^16^ revealed that forced expression of mutant HRAS^Q61R^ resulted in a significant increase in the number of spheres than forced expression of EV or HRAS^WT^ in all cell lines tested (Supplementary Fig. 4c, *P* < 0.05, unpaired *t-*tests).

### *HRAS*^Q61R^ induces partial myoepithelial differentiation

**Fig. 6:**
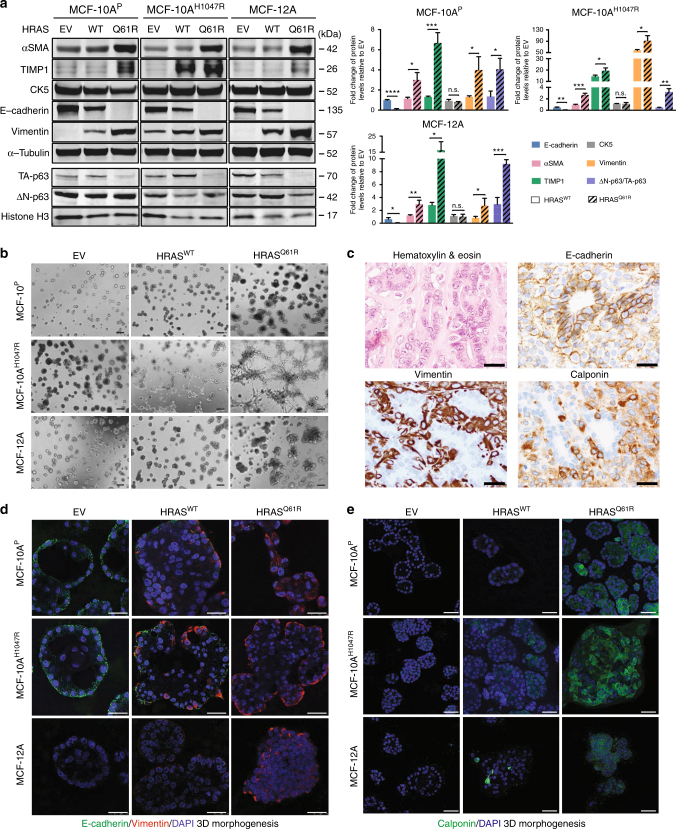
Expression of mutant HRAS^Q61R^ results in the acquisition of a partial myoepithelial phenotype in non-malignant breast epithelial cells. **a** Representative western blot (left) analysis of total protein expression of alpha-smooth muscle actin (αSMA), TIMP1, cytokeratin 5 (CK5), E-cadherin, vimentin, and nuclear protein expression of ∆N-p63 and TA-p63 in MCF-10A^P^, MCF-10A^H1047R^, and MCF-12A cells stably expressing empty vector (EV), HRAS^WT^, or mutant HRAS^Q61R^. α-Tubulin and Histone H3 were used as protein loading controls for total and nuclear protein expression, respectively. Quantification (right) using LI-COR is shown based on experiments done in triplicate. Comparisons of protein levels were performed between HRAS^WT^ and mutant HRAS^Q61R^, both relative to EV. Error bars, s.d. of mean (*n* = 3). n.s. = not significant, *P < 0.05, **P < 0.01, ***P < 0.001, ****P < 0.0001; two-tailed unpaired *t*-test. **b** Representative micrographs of cells cultured in three-dimensional basement membrane for 10 days showing the effects of EV, HRAS^WT^, or mutant HRAS^Q61R^ expression in MCF-10A^P^, MCF-10A^H1047R^, and MCF-12A cells on growth and glandular architecture (scale bars, 400 µm). **c** Representative micrographs of E-cadherin, vimentin, and calponin immunohistochemical expression in a *HRAS* and *PIK3CA* mutant adenomyoepithelioma (AM32). Note the bi-layered glandular architecture where E-cadherin is preferentially expressed in the inner epithelial layer, whereas vimentin and calponin decorate the outer myoepithelial layer (scale bars, 50 µm). **d**, **e** Representative confocal images of immunofluorescence analysis of **d** E-cadherin (green) and vimentin (red) and 4,6-diamidino-2-phenylindole (DAPI, blue; scale bars, 25 µm), and **e** calponin (green) and DAPI (blue; scale bars, 50 µm) of MCF-10A^P^, MCF-10A^H1047R^, and MCF-12A cells stably expressing EV, HRAS^WT^, or mutant HRAS^Q61R^ grown in three-dimensional basement membrane culture for 10 days. In **b**, **d** and **e**, experiments were independently performed at least three times

Functional experiments provided evidence that the *HRAS* Q61R mutation with or without a *PIK3CA* H1047R or E545K mutation results in increased proliferation and migration in non-malignant breast epithelial cells. Given that *HRAS* Q61 hotspot mutations are vanishingly rare in common forms of breast cancers (Fig. 3b**)**, but present in the majority of ER-negative adenomyoepitheliomas and always in conjunction with *PIK3CA* or *PIK3R1* somatic mutations, we posited that *HRAS* Q61 hotspot mutations would not only constitute an oncogenic driver of ER-negative adenomyoepitheliomas, but also play a role in the acquisition of an adenomyoepithelial phenotype. To determine the impact of *HRAS* and *PIK3CA* hotspot mutations on the differentiation of non-malignant breast epithelial cells, we assessed the expression levels of proteins preferentially expressed in epithelial or basal/myoepithelial cells of the breast^17,18^ in MCF-10A^P^, MCF-10A^H1047R^, MCF-10A^E545K^, and MCF-12A cells grown in monolayer, and the phenotypic changes induced by *HRAS* and *PIK3CA* hotspot mutations when MCF-10A^P^, MCF-10A^H1047R^, and MCF-12A cells were grown in three-dimensional model systems (Fig. 6).

In monolayer cultures, forced expression of mutant HRAS^Q61R^ in MCF-10A^H1047R^, MCF-10A^E545K^, and MCF-12A resulted in downregulation of E-cadherin, which is not expressed in normal myoepithelial cells^19^, and upregulation of alpha-smooth muscle actin (αSMA), an integral component of the smooth muscle apparatus of myoepithelial cells^18^ and expressed in adenomyoepitheliomas^1^, TIMP1, another marker of myoepithelial differentiation^18^, and vimentin, which is also expressed in myoepithelial cells of normal breast^18^ and adenomyoepitheliomas^2^, as compared to EV or forced expression of HRAS^WT^ (Fig. 6a, Supplementary Fig. 5d). In addition, forced expression of mutant HRAS^Q61R^ led to a significantly increased ∆N-p63 (p40)/TA-p63 ratio as compared to EV or forced expression of HRAS^WT^ (Fig. 6a, Supplementary Fig. 5d, *P* < 0.05, unpaired *t-*tests). It should be noted that ∆N-p63 is the p63 isoform preferentially expressed in cells with myoepithelial differentiation^20,21^, whereas TA-p63 is constitutively expressed at baseline in MCF-10A cells^22^ and reported to have anti-suppressive properties^23^. Consistent with the notion that this phenomenon is related to the acquisition of a partial myoepithelial phenotype rather than epithelial-to-mesenchymal transition, the levels of cytokeratin 5 (CK5), which is expressed in epithelial and myoepithelial cells of normal breast and adenomyoepitheliomas^1^, did not change accordingly (Fig. 6a, Supplementary Fig. 5d). Quantitative real-time reverse transcription PCR (qRT-PCR) confirmed the significantly higher levels of genes preferentially expressed in normal myoepithelial cells of the breast^17,18^, including *ACTA2* (encoding αSMA)^1^, *TIMP1,* *SPARC*, and *VIM* (encoding vimentin), and significant downregulation of *CDH1* (encoding E-cadherin) in MCF-10A^P^, MCF-10A^H1047R^, and MCF-12A cells expressing mutant HRAS^Q61R^ as compared to cells expressing HRAS^WT^ (Supplementary Fig. 4d, *P* < 0.05, unpaired *t-*tests). These observations suggest that forced expression of mutant HRAS^Q61R^ may be sufficient to induce the acquisition of at least a partial myoepithelial phenotype in non-malignant breast epithelial cells.

Forced expression of mutant HRAS^Q61R^ in MCF-10A^P^, MCF-10A^H1047R^, and MCF-12A cells grown in three-dimensional organotypic cultures^24,25^ resulted in a phenotype shift; from round, regular, polarized acinar structures with hollow lumina to irregular, multi-acinar structures connected through duct-like extensions with partially filled lumina (Fig. 6b), a phenotype reminiscent of myoepithelial colonies grown in Matrigel^26^. Akin to human adenomyoepitheliomas that display markedly decreased E-cadherin expression in the myoepithelial cells as compared to the epithelial cells and express vimentin in the myoepithelial cell layer (Fig. 6c), immunofluorescence analysis of the acinar structures of MCF-10A^P^, MCF-10A^H1047R^, and MCF-12A cells demonstrated that forced expression of wild-type and mutant HRAS resulted in downregulation of E-cadherin and upregulation of vimentin (Fig. 6d). Importantly, however, in MCF10A^H1047R^ and MCF12A cells, forced expression of mutant HRAS^Q61R^ resulted in vimentin expression in the abluminal layer, closely recapitulating its expression in the gland-like structures found in human adenomyoepitheliomas (Fig. 6c). In addition, forced expression of mutant HRAS^Q61R^ led to a consistent increase in the expression levels of calponin, another marker of myoepithelial differentiation^18,27^ expressed in adenomyoepitheliomas^1^, in all cell lines tested (Fig. 6e). qRT-PCR analysis of RNA extracted from acinar structures corroborated the downregulation of *CDH1* and upregulation of *SPARC* and *TIMP1* (Supplementary Fig. 4e).

Taken together, these observations are consistent with the hypothesis that HRAS^Q61R^ may be sufficient for the acquisition of a partial myoepithelial phenotype in ER-negative non-malignant breast epithelial cells, and that this phenotype becomes more overt in the presence of a *PIK3CA* H1047R hotspot mutation.

### *HRAS*^Q61R^ induces strong activation of the PI3K-AKT pathway

Co-occurrence of genetic alterations that induce activation of MAPK and PI3K-AKT pathways has been shown to result in stronger oncogenic potential than genetic alterations affecting either pathway alone^28^. Given that cross-talks between both pathways occur and RAS is a positive regulator of AKT^28,29^, we sought to investigate whether *HRAS* Q61 mutations are preferentially acting via the PI3K-AKT or MAPK signaling cascades, and the effects of AKT (MK2206) and MEK (GSK212) pharmacological inhibition in HRAS^Q61R^-expressing MCF-10A^P^ and MCF-10A^H1047R^ cells.

**Fig. 7:**
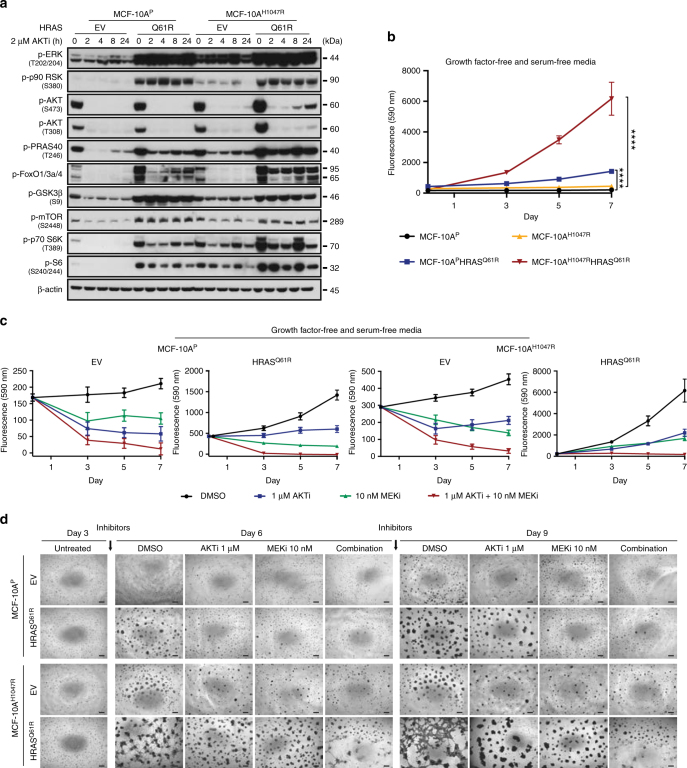
Impact of AKT and MEK inhibition on PI3K-AKT and MAPK signaling pathways and proliferation in non-malignant breast epithelial cells expressing mutant HRAS^Q61R^. **a** Representative western blot analysis of p-ERK1/2 (T202/Y204), p-p90 RSK (S380), p-AKT (S473), p-AKT (T308), p-PRAS40 (T246), p-FOXO1/3a/4, p-GSK3β (S9), p-mTOR (S2448), p-p70 S6K (T389), and p-S6 (S240/244) protein in MCF-10A^P^ and MCF-10A^H1047R^ cells stably expressing empty vector (EV) or mutant HRAS^Q61R^ treated with 2 µM AKT inhibitor (AKTi, MK2206) at different time points. β-actin was used as a protein loading control. Experiments were repeated at least twice with similar results**. b** Cell proliferation assay of MCF-10A^P^ and MCF-10A^H1047R^ cells stably expressing EV or mutant HRAS^Q61R^. ****P *<* 0.0001; two-tailed unpaired *t*-test. **c** Inhibition effects of cells treated with DMSO (black), 1 µM AKTi (blue), 10 nM MEK inhibitor (MEKi, GSK212, green), and combination of 1 µM AKTi and 10 nM MEKi (red) for 3, 5, and 7 days. In **b** and **c**, cells were cultured in growth factor- and serum-free media. Data are representative of three independent experiments. Error bars, s.d. of mean (*n* = 3). **d** Representative micrographs of MCF-10A^P^ and MCF-10A^H1047R^ cells stably expressing EV or mutant HRAS^Q61R^ cultured after 3 days and treated with DMSO, 1 µM AKTi, 10 nM MEKi, or combination of 1 µM AKT and 10 nM MEK inhibitors for 6 and 9 days are shown (scale bars, 400 µm). DMSO or inhibitors were added after seeding the cells in three-dimensional basement membrane for 3 days; fresh media with DMSO or inhibitors was replenished every 3 days. Triplicate experiments were repeated at least twice with similar results

We first assessed the phosphorylation of the downstream targets of RAS and AKT at baseline and upon treatment with AKT and MEK inhibitors (AKTi and MEKi, respectively) at different time-points (Fig. 7a, Supplementary Fig. 6a). Forced expression of HRAS^Q61R^ as compared to EV resulted in higher phosphorylation of MAPK signaling pathway components, as well as of markers downstream of AKT and mTOR, not only in MCF-10A^P^, but also in MCF-10A^H1047R^ cells (Fig. 7a, Supplementary Fig. 6a). These findings suggest that activation of PI3K-AKT-mTOR pathway is a key consequence of the *HRAS* Q61R mutation, and that both *HRAS* Q61 and *PIK3CA* mutations may cooperate for strong activation of the PI3K-AKT-mTOR pathway in ER-negative adenomyoepitheliomas.

Next, we conducted dose−response studies to assess the dependency of RAS-induced proliferation on MAPK or AKT signaling (Supplementary Fig. 6b). The median inhibitory concentrations (IC50s) observed suggest that MCF-10A^P^ and MCF-10A^H1047R^ cells with forced expression of mutant HRAS^Q61R^ were less sensitive to AKT inhibition than those cells expressing EV, whereas EV- and mutant HRAS^Q61R^-expressing MCF-10A^P^ and MCF-10A^H1047R^ cells showed similar sensitivity to MEK inhibition (Supplementary Fig. 6c). In growth factor- and serum-free media, forced expression of mutant HRAS^Q61R^ in MCF-10A^P^ and MCF-10A^H1047R^ cells revealed a significant increase in cell proliferation (Fig. 7b, *P* < 0.0001, unpaired *t-*test), which was inhibited in part by either AKT or MEK inhibition regardless of the *PIK3CA* status. Importantly, however, the combination of both inhibitors completely abrogated cell proliferation (Fig. 7c).

To assess the effects of AKT and MEK inhibition further, we treated EV- or mutant HRAS^Q61R^-expressing MCF-10A^P^ and MCF-10A^H1047R^ cells grown in three-dimensional cultures. AKT and MEK inhibition led to a partial reversion of the phenotypic transformation caused by mutant HRAS^Q61R^ expression in MCF-10A^P^ and MCF-10A^H1047R^ cells, which was more overt under treatment with the combination of AKT and MEK inhibitors (Fig. 7d), consistent with the results obtained in monolayer cultures.

Taken together, these findings demonstrate that *HRAS*^Q61R^ induces strong activation of the PI3K-AKT pathway in non-malignant breast epithelial cells; however, both PI3K-AKT and MAPK pathways likely contribute to RAS-mediated proliferation in these cells.

## Discussion

Here we demonstrate that breast adenomyoepitheliomas constitute a heterogeneous group of tumors, characterized by recurrent pathogenic somatic mutations in *HRAS* and PI3K-AKT pathway genes. We further show that the histologic and genetic features of adenomyoepitheliomas vary according to ER status: ER-negative adenomyoepitheliomas more frequently display histologic features associated with an aggressive clinical behavior and frequently harbor concurrent mutations in *HRAS* Q61 and *PIK3CA* or *PIK3R1* (56%), whereas ER-positive adenomyoepitheliomas are largely underpinned by *PIK3CA* or *AKT1* mutations (59%). Members of the PI3K-AKT pathway, including *PIK3CA, PIK3R1*, and *AKT1*, are frequently affected by somatic genetic alterations in breast cancer^6^ and breast cancer precursor lesions^30^. By contrast, *HRAS* Q61 hotspot mutations are vanishingly rare in breast cancer^6,7^, suggesting that in the breast, the co-occurrence of *HRAS* Q61 and PI3K pathway gene mutations may be associated with an adenomyoepithelial phenotype. In fact, the sole *HRAS* Q61 mutation found in the TCGA and ICGC breast cancer datasets was present in a high-grade triple-negative breast cancer, which was *TP53* wild-type and harbored both an *HRAS* Q61L and a *PIK3CA* H1047R mutations^7^. One could posit that this invasive breast cancer may have constituted a triple-negative invasive carcinoma arising from an adenomyoepithelioma.

*HRAS* and PI3K-AKT pathway mutations identified by massively parallel sequencing were found to be clonal in the vast majority of cases and truncal in the three cases subjected to sequencing analysis of different components (i.e., primary adenomyoepithelioma, carcinoma and/or metastatic lesions), consistent with the notion that these mutations likely constitute founder genetic events in the development of adenomyoepitheliomas. Given that two cases harbored clonal *HRAS* mutations but subclonal *PIK3CA* or *PIK3R1* mutations (Supplementary Fig. 2), one could posit that the *HRAS* hotspot mutations may precede the mutations affecting PI3K pathway genes in the development of ER-negative adenomyoepitheliomas. The acquisition of additional genetic changes, such as *TERT* promoter mutations and *CDKN2A* homozygous deletions, may play a role in tumor progression. In fact, an exploratory, hypothesis-generating analysis of *TERT* gene promoter mutations and *CDKN2A* homozygous deletions in the adenomyoepitheliomas subjected to massively parallel sequencing revealed a significant association with the presence of a carcinoma (Fig. 2, *TERT* gene promoter mutations, *P* = 0.0307, and *CDKN2A* homozygous deletions, *P* = 0.0086, Fisher’s exact tests). Further larger studies are warranted to test whether the presence of *TERT* gene promoter mutations and of loss of p16 protein expression (i.e., the protein product of *CDKN2A*) may predict the behavior of ER-negative adenomyoepitheliomas.

In vitro analyses demonstrated that forced expression of mutant HRAS^Q61R^ alone or in the presence of mutant *PIK3CA*^H1047R^ or *PIK3CA*^E545K^ results in oncogenic properties and acquisition of a myoepithelial-like phenotype in non-malignant, *TP53* wild-type, ER-negative breast epithelial cells. Albeit not necessary for the acquisition of a myoepithelial phenotype, given that adenomyoepitheliomas lacking *HRAS* mutations were observed in this study, forced expression of HRAS^Q61R^ was sufficient to induce a partial myoepithelial phenotype. Depending on the context and culture conditions, myoepithelial differentiation was more pronounced in cells harboring a knocked-in *PIK3CA*^H1047R^ mutation than in *PIK3CA*^WT^ cells, suggesting that these mutations may cooperate in the development and/or maintenance of the adenomyoepithelioma phenotype. In fact, our data suggest that mutant HRAS results in strong activation of the PI3K-AKT-mTOR signaling cascade, enhancing its signaling in a *PIK3CA-*mutant background (Fig. 7a, Supplementary Fig. 6a). Finally, given that the *PIK3CA* H1047R hotspot mutation, when introduced in the basal/myoepithelial compartment of mouse mammary glands, induces the development of tumors that often recapitulate adenomyoepitheliomas^31^, further studies are required to define whether the likely cell of origin of most ER-negative breast adenomyoepitheliomas would reside in the basal compartment of the mammary gland (where progenitor cells likely reside^32^), as opposed to the luminal progenitor compartment, the origin of the vast majority of breast cancers, including those of basal-like and triple-negative phenotype^33^. Consistent with this notion, MMTV-Wnt1 mice have been shown to develop tumors that likely originate from progenitor cells^34^ and display several characteristics that resemble those of human ER-negative adenomyoepitheliomas, including a basal/myoepithelial transcriptomic profile^35^, histologic features similar to those of adenomyoepitheliomas, and *Hras* Q61 mutations as a somatic event in up to 37% of cases, exclusively in *Trp53* wild-type lesions^36^. Conversely, the relevance of the MMTV-H-Ras mouse model for the study of adenomyoepitheliomas remains to be fully determined, given that these animals express wild-type rather than mutant *Hras* and have histologic features that appear to be distinct from those of human adenomyoepitheliomas^37^, highlighting that caution should be exercised in the translation of the genotypic and phenotypic characteristics of mouse mammary gland tumors to human breast neoplasms.

We and others have demonstrated that rare tumors that originate in distinct anatomical sites are not uncommonly underpinned by highly recurrent somatic genetic alterations (e.g., adenoid cystic carcinomas, which are driven by the *MYB-NFIB* or *MYBL1* rearrangements in the breast, salivary glands and the lungs)^38,39^. Adenomyoepitheliomas, in particular those displaying an ER-negative phenotype, bear a striking histologic similarity with epithelial−myoepithelial carcinomas of the salivary glands. Consistent with the notion that ER-negative adenomyoepitheliomas may constitute the breast counterpart of epithelial−myoepithelial carcinomas of the salivary glands, up to 80% of salivary gland epithelial−myoepithelial carcinomas have been reported to harbor *HRAS* Q61 hotspot mutations^40,41^, which are reported to co-occur with *PIK3CA* mutations in approximately 40% of cases^40^. Taken together, the genomic analyses presented here and in previous studies^40,41^, and the in vitro data generated here suggest a potential genotypic−phenotypic association, where *HRAS* Q61 mutations in conjunction with *PIK3CA* or *PIK3R1* mutations may result in the development of tumors with an epithelial−myoepithelial phenotype in certain anatomical sites.

Our study has several limitations. Given that cases were obtained from multiple institutions and consultation files of the authors, we could not perform a systematic analysis of the impact of specific somatic genetic alterations on the outcome of patients with adenomyoepithelioma. A subset of adenomyoepitheliomas lacked mutations in *HRAS* and/or PI3K-AKT pathway genes, one of which harbored an *ERBB3* hotspot mutation. Further studies are warranted to define the genomic drivers of adenomyoepitheliomas lacking *HRAS* and PI3K-AKT pathway gene mutations. Although we demonstrated here that the *HRAS* Q61R hotspot mutation, in particular in association with the *PIK3CA* hotspot mutations, may be sufficient for the acquisition of a myoepithelial phenotype in non-malignant breast epithelial cells and induces AKT signaling, the mechanistic basis for the acquisition of this myoepithelial differentiation program has yet to be defined. This would be ideally achieved using patient-derived adenomyoepithelioma cell lines, xenografts, and organoids, which are currently not commercially available. Finally, given that the *PIK3CA* H1047R hotspot mutation, when introduced in the basal/myoepithelial compartment of mouse mammary glands, induces the development of tumors that often recapitulate adenomyoepitheliomas^31^, further studies are required to define whether the likely cell of origin of most ER-negative breast adenomyoepitheliomas would reside in the basal compartment of the mammary gland.

In conclusion, breast adenomyoepitheliomas are heterogeneous and genomically distinct on the basis of their ER status. *HRAS* Q61 hotspot mutations and mutations affecting PI3K-AKT pathway-related genes likely constitute drivers of these tumors. The *HRAS* Q61R hotspot mutation was found to promote the acquisition of the cardinal features of ER-negative adenomyoepitheliomas in in vitro models, in particular in the presence of a *PIK3CA* H1047R or E545K hotspot mutation. Our findings contextualize the biological significance of *HRAS* Q61 hotspot mutations in the realm of breast neoplasms, and illustrate genotypic-phenotypic association in the taxonomy of breast tumors.

## Methods

### Sample selection and ethics

After obtaining approval by the IRBs and the local research ethics committees from the authors’ institutions, representative histologic formalin-fixed paraffin-embedded (FFPE) blocks of 53 adenomyoepitheliomas of the breast were retrieved from the archives of the authors’ institutions. Patient consent was obtained where appropriate, according to the IRB-approved protocols. Samples were anonymized prior to the analyses. After central histologic review by five pathologists with expertise in breast pathology (F.C.G., M.E., I.O.E., E.A.R., and J.S.R.-F.), 43 cases were unanimously diagnosed as breast adenomyoepithelioma following the World Health Organization classification (WHO) criteria^2^, and were included in this study (Supplementary Data 1). With an estimated mutation rate of 20 non-synonymous somatic mutations affecting protein coding genes per case and using Sidak correction (5% overall error rate), a sample size of 43 would confer 80% power for the detection of a recurrent genetic alteration if its true incidence is ≥15%. This study is compliant with the Declaration of Helsinki.

Histologic assessment of architectural subtype, necrosis, mitotic rate, nuclear pleomorphism, and associated carcinomas were performed by three pathologists (F.C.G., F.P., and J.R.S.-F.). The architectural subtype was defined as tubular or papillary following previously defined criteria^1,2^. Necrosis, mitotic rate, and nuclear pleomorphism have been shown to constitute histologic features associated with aggressive behavior and malignant transformation in breast adenomyoepitheliomas^1,2^. Necrosis was defined as present (any area) or absent. Mitotic rate was defined as the number of mitotic figures in the myoepithelial or epithelial cell compartments per mm^2^, and stratified into three categories. Nuclear pleomorphism was evaluated according to the Nottingham histologic grading system of breast cancer^42^. The presence and histologic type of an associated invasive carcinoma in the primary adenomyoepitheliomas and/or recurrent lesions in the ipsilateral breast was assessed according to the WHO criteria^2^.

### Immunohistochemistry

Representative 4-μm-thick FFPE tumor sections of each case were subjected to immunohistochemical analysis with antibodies against ER (prediluted, clone 6F11, antigen retrieval ER1 solution for 20 min, Leica), and selected cases were immunohistochemically analyzed for the expression of E-cadherin (prediluted, clone 36, antigen retrieval CC1 for 16 min, Ventana), vimentin (prediluted, clone V9, antigen retrieval CC1 for 32 min, Ventana), HER2 (prediluted, 4B5, CC1 for 20 min, Ventana), p63 (prediluted, clone 4A4, antigen retrieval CC1 for 30 min, Ventana), p53 (prediluted, clone DO-7, antigen retrieval CC1 for 27 min, Ventana), Ki67 (1:400, clone MIB-1, antigen retrieval CC1 for 30 min, Dako), AR (1:500, clone SP107, antigen retrieval CC1 for 30 min, Ventana) and calponin (prediluted, clone EP798Y, antigen retrieval CC1 for 16 min, Ventana). Positive and negative controls were included in each slide run. The ER and HER2 status was defined according to the American Society of Clinical Oncology (ASCO)/College of American Pathologists (CAP) guidelines^43,44^. The expression of E-cadherin, vimentin, calponin, and p63 was analyzed as previously described^45^. AR expression was defined as positive when ≥1% of tumor cells displayed nuclear expression; for statistical comparisons, we used a cut-off of ≥10% that has been previously employed to select patients for anti-androgen therapy in an early-phase clinical trial^11^.

### Microdissection and nucleic acid extraction

Eight-µm-thick sections of FFPE blocks representative of the tumor and normal tissues (i.e. unaffected lymph nodes or breast tissue devoid of terminal duct-lobular units away from the tumor site) were stained with nuclear fast red and microdissected using a sterile needle under a stereomicroscope (Olympus SZ61), to ensure a tumor cell content >80% and that the normal tissue was devoid of any neoplastic cells as previously described^46^. DNA extraction from microdissected tumor samples and normal adjacent tissues was performed separately using the DNeasy Blood and Tissue Kit (Qiagen), according to the manufacturer’s guidelines. We obtained DNA of sufficient quantity and quality of 10, 21, and 12 samples of primary tumors for WES, MSK-IMPACT and Sanger sequencing only, respectively (Supplementary Data 1). In addition, we obtained sufficient DNA from separately microdissected components of the primary tumor, local recurrence in the breast tissue, and/or metastases for three cases (AM5, AM8, and AM46). DNA quantity and quality were analyzed using a Qubit Fluorometer (Invitrogen, ThermoFisher) and a TapeStation (Agilent), respectively.

### WES and MSK-IMPACT massively parallel sequencing

WES and MSK-IMPACT, a massively parallel sequencing assay targeting all exons and selected non-coding and regulatory regions of 410 key cancer genes, were performed at the Memorial Sloan Kettering Cancer Center (MSKCC) Integrated Genomics Operation (IGO) on matched tumor and normal DNA samples from 10 and 21 adenomyoepitheliomas, respectively (Supplementary Data 1), as previously described^47,48^.

Analyses of the sequencing data and the detection of somatic mutations and allele-specific CNAs were performed exactly as previously described^47,48^. In brief, reads were aligned to the human reference genome GRCh37 using the Burrows−Wheeler Aligner^49^. Local realignment, de-duplication, and quality score recalibration were performed using the Genome Analysis Toolkit (GATK)^50^. Somatic single nucleotide variants (SNVs) were identified using MuTect^51^; small insertions and deletions (indels) were identified using Strelka and VarScan 2^52,53^, and further curated by manual inspection. Variants found with >5% global minor allele frequency in dbSNP (Build 137) or that were covered by <10 reads in the tumor or <5 reads in the germline were disregarded^47,54^. Variants for which the tumor variant allele fraction was <5 times that of the normal variant allele fraction were disregarded^47,54^. We adopted this conservative approach to minimize false positive results obtained with DNA extracted from FFPE samples^47^. The potential functional effect of each missense SNV was investigated following our previously described approach^48,55^, using a combination of benchmarked mutation effect algorithms^56^. Hotspot mutations were annotated according to Chang et al.^57^. Allele-specific CNAs were inferred from WES or MSK-IMPACT data using FACETS^58^ as previously described^48,55^. In brief, read counts for dbSNP (build 137) positions within the target regions with dbSNP entries (build 137) were generated for matched tumor and normal samples, and used as input to FACETS, which performs a joint segmentation of the total and allelic copy ratio and infers allele-specific copy number states, using the following parameters: pre-processing critical value (Pre CVAL) 50, critical value for estimating diploid Log_2_ ratio (CVAL1) 150, critical value for segmentation (CVAL2) 50, and minimum number of heterozygous SNPs in a segment used for bivariate t-statistic during clustering of segment (Min Nhet) 25. Genes with CNAs were determined adopting the methods described in Curtis et al.^59^ and in the supplementary materials of Piscuoglio et al.^47^. The cancer cell fraction (CCF) of each mutation was inferred using ABSOLUTE (v1.0.6)^12^, as previously described^47,48,55^. Solutions from ABSOLUTE were manually reviewed as described^12,60^. A mutation was classified as clonal if its probability of being clonal was >50%^60^ or if the lower bound of the 95% confidence interval of its CCF was >90%^61^. Mutations that did not meet the above criteria were considered subclonal. Phylogenetic trees were generated using Treeomics^62^, using the CCF values and the depth as input.

### Validation of mutations by targeted amplicon re-sequencing

A random subset of 66 somatic mutations, encompassing 78% of the non-synonymous mutations identified by WES in samples AM1−AM7 (excluding case AM5 and its associated lesions, see below) were validated by targeted amplicon re-sequencing using a custom Ion Torrent AmpliSeq panel. Sequencing was performed to a median depth of 832× (range 560×−941×) and 664× (range 257×−949×) for the tumor and germline samples, respectively. Paired-end reads in FASTQ format were aligned to the reference human genome GRCh37 using the Torrent Mapping Alignment Program (TMAP, v3.4.1)^63^. Local realignment was performed using GATK (v3.1.1)^50^. Putative mutations were interrogated using pileup files generated with samtools mpileup (version 1.2 htslib 1.2.1)^64^. Mutations present at variant allele frequencies >1% were considered “validated”. Mutations that did not validate were excluded from further analyses. Given the overall validation rate of 92.4% (Supplementary Data 2), mutations that were not tested were included in the final results. For AM5 and its associated lesions, validation was performed through independent WES assays from new libraries generated from the original DNA samples of each lesion; only mutations found in both WES assays from each component were considered as validated.

### Sanger sequencing

For validation of the *HRAS, PIK3CA*, and *AKT1* mutations detected in the samples subjected to WES or MSK-IMPACT sequencing and for the screening of mutations affecting these genes in additional 12 samples not subjected to massively parallel sequencing, PCR amplification and Sanger sequencing were performed as previously described^47,48^ (primer sequences upon request). In addition, the *TERT* gene promoter was assessed either by MSK-IMPACT or Sanger sequencing, using the primers and methods described in Piscuoglio et al.^47^. Sequencing reactions were performed in triplicate, and both the forward and reverse strands were analyzed using MacVector software.

### Antibodies and small molecule inhibitors

For western blotting, we employed antibodies against HRAS (Santa Cruz Biotechnology sc520, rabbit, 1:500), αSMA (Abcam ab5694, rabbit, 1:500), CK5 (ThermoFisher MA5-17057, mouse, 1:500), and p63 (Biolegend 619002, rabbit, 1:500), and the following antibodies purchased from Cell Signaling Technology (CST): rabbit anti-p-MEK (S217/221; 9154, 1:1000), anti-p-ERK1/2 (T202/Y204; 4370, 1:1000), anti-ERK1/2 (9107, 1:1000), anti-p-p90 RSK (S380; 9341, 1:1000), anti-p-AKT (S473; 4060, 1:1000), anti-p-AKT (T308; 2965, 1:1000), anti-AKT (9272, 1:1000), anti-p-PRAS40 (T246; 13175, 1:1000), anti-p-FOXO1/3a/4 (2599, 1:1000), anti-p-GSK3β (S9; 5558, 1:1000), anti-p-mTOR (S2448;5536, 1:1000), anti-p-p70 S6K (T389; 9205, 1:1000), anti-p-S6 (S240/244; 5364, 1:1000), anti-p-4EBP1 (S65; 9451, 1:1000), anti-p-4EBP1 (T37/46; 2855, 1:1000), anti-TIMP1 (8946, 1:1000), anti-vimentin (5741, 1:1000), anti-α-Tubulin (2125, 1:1000), anti-β-actin (4970, 1:1000) and anti-Histone H3 (4499, 1:1000), and mouse anti-E-cadherin (14472, 1:1000) and anti-α-Tubulin (3873, 1:1000). For immunofluorescence, antibodies against vimentin (CST 5741, rabbit, 1:100), E-cadherin (CST 14472, mouse, 1:50), and calponin (Abcam ab46794 rabbit, 1:100) were used.

The AKT inhibitor MK2206 (MedChem Express HY-10358) and MEK inhibitor GSK212 (Selleck Chemicals s2658) were employed for pharmacological inhibition in cell lines analyzed in in vitro experiments time points and concentrations indicated.

### Mutagenesis and vector and stable cell line generation

The human HRAS (NM_005343) cDNA ORF clone was purchased from Origene (RG216409), and the Q61R mutation was introduced using the Q5 Site-Directed Mutagenesis Kit (New England Biolabs E0554) following the manufacturer’s recommendations. HRAS^WT^ and mutant HRAS^Q61R^ open reading frames were cloned into the pLenti-EF1a-GFP-2A-Puro vector (ABM LV067) and pcDNA™3.3-TOPO^®^ vector (ThermoFisher K830001) for stable and transient transfection, respectively. Sanger sequencing was used to confirm the reading frames of HRAS^WT^ and HRAS^Q61R^. Human isogenic MCF-10A *PIK3CA* wild-type (MCF-10A^P^) and MCF-12A cells were purchased from ATCC. *PIK3CA* H1047R mutant (MCF-10A^H1047R^) and E545K mutant (MCF-10A^E545K^) cells were purchased from Horizon (X-MAN). Cell lines were authenticated by short tandem repeat profiling as previously described^65^, and tested for mycoplasma infection using the Universal Mycoplasma Detection Kit (ATCC). Cell lines were cultured as previously described^66^. Transfections of EV, HRAS^WT^, and mutant HRAS^Q61R^ were performed as previously described^54,66^, using Lipofectamine™ LTX Reagent (ThermoFisher 15338100) according to the manufacturer’s protocol. For stable selection, transfected cells were selected with 1 µg/ml puromycin. Resistant colonies formed at 15−20 days of selection.

### HRAS^WT^ and mutant HRAS^Q61R^ activation assay

Pull-down of the active form of HRAS from stable EV, HRAS^WT^, and mutant HRAS^Q61R^ MCF-10A^P^, MCF-10A^H1047R^, and MCF-12A cell lysates was performed using two independent active HRAS pull-down and detection kits from Cell Biolabs, Inc. (STA-400-H-T) and ThermoFisher (16117), respectively. Briefly, 80–90% confluent cells from each group were scraped and collected using the lysis/wash buffer provided. 40 µl to 80 µl of resuspended GST-Raf1-RBD Agarose beads were mixed into each cell lysate (containing at least 500 µg of total protein) and incubated at 4 °C for 1 h followed by three washes with 0.5 ml of lysis/wash buffer. Subsequently, the bead pellet was resuspended in 40−50 µl of 2× reducing SDS-PAGE sample buffer (β-mercaptoethanol-2× SDS sample buffer 1:20). After centrifugation, the eluted samples were boiled for 5 min and loaded onto a polyacrylamide gel for GTP-HRAS detection by western blot analysis using an anti-HRAS antibody.

### Western blotting

Total protein and nuclear protein lysates were prepared using the M-PER Mammalian Protein Extraction Reagent and NE-PER Nuclear and Cytoplasmic Extraction Reagents, respectively, supplemented with Halt Protease and Phosphatase inhibitors cocktail (ThermoFisher). Standard western blotting was conducted as previously described^66^. Membranes were probed with primary antibodies and followed by incubation with HRP-tagged (CST 7074) or conjugated IRDye 680RD/800CW (LI-COR Biosciences) secondary antibodies, visualized on a Syngene ChemiGenius with Super-Signal West Dura Chemiluminescence Substrate (Pierce) or the Odyssey Infrared Imaging System, and quantified by the LI-COR Image Studio Software. When required, the membranes were stripped using the Restore PLUS Western Blot Stripping Buffer (ThermoFisher). Replicate experiments were performed as indicated. Unprocessed scans of blots are available in Supplementary Figs. 7−10.

### Colony formation assay

Soft agar colony formation assay was performed as previously described^54,66^. Briefly, MCF-10A^P^, MCF-10A^H1047R^, and MCF-12A stably expressing EV, HRAS^WT^, and mutant HRAS^Q61R^ (5×10^4^) were added to 1.5 ml of complete growth media with 0.4% UltraPure™ Agarose (ThermoFisher) and layered onto a 2 ml bed of complete growth media plus 0.5% of agarose. Cells were fed every 3 days with 1 ml of complete growth media. At day 21, growth media was removed and viable colonies were stained with 0.005% Crystal Violet solution (Sigma-Aldrich). Colony size was determined using Fiji (ImageJ). Size (in pixels) was measured using Feret diameter (*D*) and minimum Feret diameter (*d*) and applying the formula (*D* x *d*2)/2 = colony volume and plotted with GraphPad Prism v_7.0a. Experiments were performed in triplicate.

### Cell proliferation/viability and dose−response assay

For cell proliferation assays, cells were seeded either in completed growth media or growth factor- and serum-free media in 96-well plates, and monitored over 4 or 7 days, as indicated. Experiments were performed in triplicate. For AKT and MEK pharmacological inhibition, cells were seeded in complete media or growth factor- and serum-free media, and inhibitors were added the following day. 25 µl of Resazurin (R&DSystems, AR002) was added to each well and incubated at 37 °C for 4 h. Absorbance was read at spectra of 560_EX_ nm/590_EM_ nm using SpectraMax M5 (Molecular Devices). Growth and inhibition curves were plotted and analyzed using GraphPad Prism v_7.0a. To determine the median inhibitory concentration (IC50) of the AKT inhibitor and MEK inhibitor, mean values of the number of cells treated with the indicated inhibitors for 4 days were plotted as percentage of inhibition against the Log concentration of inhibitors (nM), and nonlinear regression analysis was performed using GraphPad Prism v_7.0a. Triplicate experiments were repeated at least twice.

### Wound-healing assay

Cells were serum starved for 16 h in 2% horse serum DMEM/F12 media without EGF, and were trypsinized, seeded (15×10^5^) and cultured overnight in six-well plates. A pipette tip was used to generate a scratch in the cell layer. Images were obtained at 0 and 24 h after scratch wounding at the same position. The percentage of wound closure indicated by scratch width reduction was assessed and plotted using GraphPad Prism v_7.0a. Experiments were performed in triplicate.

### Quantitative real-time reverse transcription PCR

Total RNA was extracted from cells grown in monolayer or three-dimensional cultures using TRIzol reagent (ThermoFisher) according to the manufacturer’s instructions. Two micrograms of the extracted total RNA from each sample were employed for cDNA synthesis using SuperScript VILO Master Mix (ThermoFisher). cDNA was amplified using the StepOnePlus Real-Time PCR System (ThermoFisher). Quantitative TaqMan RT-PCR (ThermoFisher) was performed for *CDH1* (Hs01023894_m1), *VIM* (Hs00958111_m1), *SPARC* (Hs00234160_m1), *TIMP1* (Hs00171558_m1), and *ACTA2* (Hs00426835_g1). Experiments were performed in triplicate, and expression data were normalized to the housekeeping gene *GAPDH* (Hs99999905_m1) and calculated as 2^–[(Ct of gene) − (Ct of GAPDH)]^.

### Three-dimensional organotypic cultures

Cells were seeded on top of growth factor-reduced reconstituted basement membrane (Matrigel, BD Biosciences) as previously described^54,66^, and subjected to immunofluorescence and mRNA expression analysis on day 10. Briefly, 50 µl or 200 µl of Matrigel was added to coat each well of an eight-well chamber slide (for IF staining and pharmacological inhibition) or a 24-well tissue culture plate (for RNA extraction), respectively. Five thousand cells resuspended in 500 µl of assay media (with 5 ng/ml EGF and 2% Matrigel) or 20,000 cells resuspended in 1 ml assay media were plated on top. For pharmacological inhibition, cells were treated with DMSO or the indicated inhibitors 3 days after seeding in drug-free media. Fresh media with/without DMSO or the indicated inhibitors was replenished every 3 days. Experiments were repeated at least twice.

### Immunofluorescence

Immunofluorescence analysis of three-dimensional organotypic cultures was performed as previously described^67^. Acinar structures in eight-well chambers were fixed and permeabilized in 4% PFA and 0.5% TritonX-100, respectively. After 1 h blocking in 10% goat serum, primary antibody was added for overnight incubation at 4 °C, and Alexa Fluor-conjugated secondary antibody (1:500) was added for 1 h incubation at room temperature. The slides were mounted in ProLong Gold antifade reagent with DAPI (ThermoFisher). Confocal analyses were performed with the Leica SP5 DM confocal microscopy system equipped with four lasers: an ultraviolet (UV) diode (405 nm), an argon laser (458, 476, 488, and 514 nm), a 543 nm HeNe laser, and a 633 nm HeNe laser. Experiments were performed in triplicate.

### Mammosphere formation assay

Tumor/mammosphere assays were performed as previously described^68^. In brief, cells were plated (30,000/well) as single cell suspensions in ultralow attachment six-well plates and grown in DMEM:F12 media (serum-free) supplemented with 20 µl/ml B27 (ThermoFisher), 20 ng/ml EGF, and 20 ng/ml bFGF. Fresh media (1 ml) was added every 3 days. Mammospheres were counted and photographed at day 10. Experiments were performed in triplicate. Colony and sphere images were documented using the phase contrast EVOS XL Imaging System (ThermoFisher). Sphere number was determined using Fiji (ImageJ).

### Statistical analysis

Fisher’s exact tests, Wilcoxon tests, and unpaired *t*-tests were used for the comparison of categorical, non-parametric, and continuous parametric variables, respectively. For the comparisons of continuous data, we assessed whether the variables were heteroscedastic and utilized appropriate statistical methods accordingly. Mutual exclusivity between *PIK3CA*, *PIK3R1,* and *AKT1* was assessed using CoMEt exact test^69^. Co-occurrence between *HRAS* mutations and *PIK3CA* or *PIK3R1* mutations was assessed using a *Z *test. Statistical analyses were carried out using R v3.1.2 or GraphPad Prism v_7.0a. Two-tailed *P* values <0.05 were considered statistically significant. For all experiments, 95% confidence intervals were adopted.

### Data availability

WES and MSK-IMPACT sequencing data have been deposited in the NCBI Sequence Read Archive under the accession numbers SRP065277 and SRP065302, respectively. The publicly available dataset from the TCGA^6^ breast cancer study was retrieved from the cBioPortal website (www.cBioPortal.org) and TCGA data portal (https://tcga-data.nci.nih.gov/docs/publications/tcga/) on 07/03/2017. Somatic mutations from samples included in the ICGC breast cancer study^7^ were extracted from the supplementary materials of Nik-Zainal et al.^7^. All other remaining data are available within the article and Supplementary Files, or available from the authors upon request.

## Electronic supplementary material

Supplementary Information

Description of Additional Supplementary Files

Supplementary Data 1

Supplementary Data 2

Supplementary Data 3

Supplementary Data 4
