# Supplementary material for: Recurrent hotspot mutations in HRAS Q61 and PI3K-AKT pathway genes as drivers of breast adenomyoepitheliomas
Source: Nat Commun. 2018 May 8;9:1816. doi: 10.1038/s41467-018-04128-5 (PMC5940840; doi:10.1038/s41467-018-04128-5)
Supplement: Supplementary file 1 — Supplementary Information [file 41467_2018_4128_MOESM1_ESM.pdf]

Supplementary Information

Recurrent hotspot mutations in *HRAS* Q61 and PI3K-AKT pathway genes as drivers of breast adenomyoepitheliomas

Geyer et al.

Supplementary Figures 1-10

SUPPLEMENTARY FIGURES

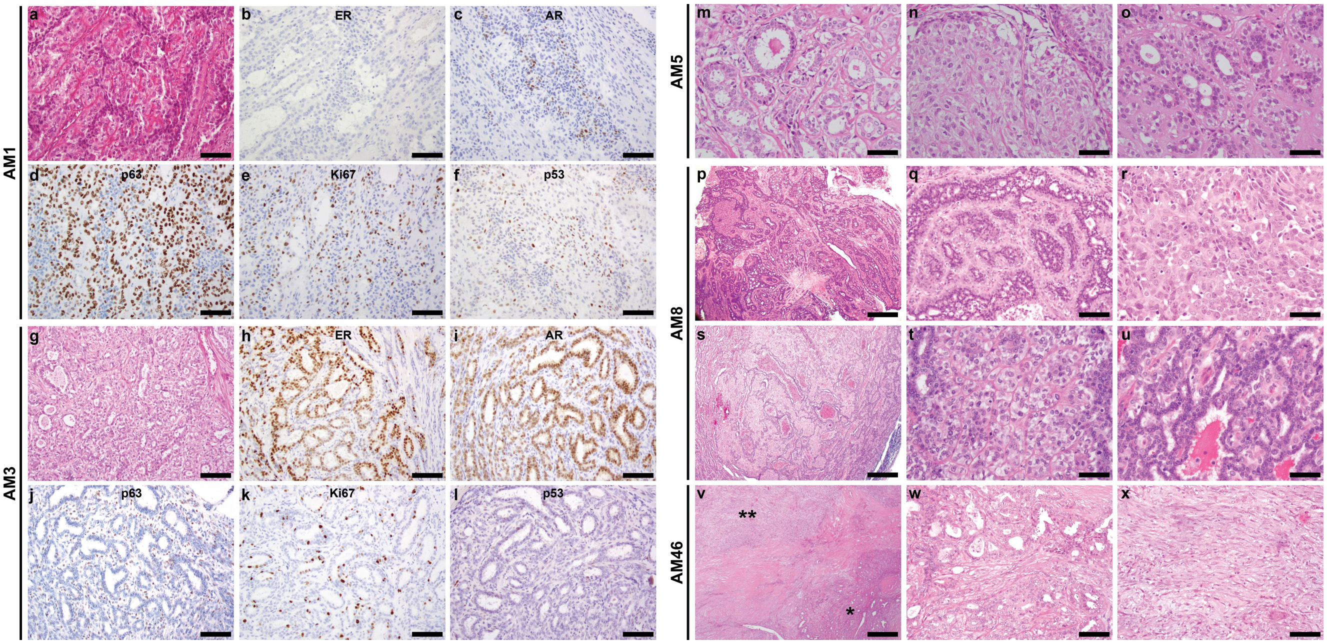

Supplementary Figure 1: Representative micrographs of breast adenomyoepitheliomas AM1 and AM3 (H&E and immunohistochemistry), and of AM5, AM8 and AM46, and their associated lesions (H&E).

a-f AM1 (a) Medium power magnification of AM1 (scale bar, 200µm), which **(b)** lacked expression of estrogen receptor (ER, scale bar, 200µm), and displayed **(c)** focal expression of androgen receptor (AR) in the epithelial cells (scale bar, 200µm), **(d)** diffuse expression of p63 in the myoepithelial cells (scale bar, 200µm), **(e)** high Ki67 expression levels (scale bar, 200µm), and **(f)** focal expression of p53 (scale bar, 200µm). **g-l AM3 (g)** Medium power magnification of AM3 (scale bar, 200µm), which **(h)** displayed diffuse expression of ER (scale bar, 200µm) and **(i)** AR in the epithelial cells (scale bar, 200µm), **(j)** diffuse expression of p63 in the myoepithelial cells (scale bar, 200µm), **(k)** intermediate Ki67 expression levels (scale bar, 200µm), and **(l)** focal expression of p53 (scale bar, 200µm). **m-o AM5 (m)** Medium power magnification of the tubular adenomyoepithelioma component (scale bar, 100µm). **(n)** Medium power magnification of the myoepithelial metaplastic carcinoma (scale bar, 100µm)**. (o)** Lymph node metastasis with biphasic architecture, characteristic of adenomyoepitheliomas (scale bar, 100µm). **p-u AM8 (p)** Low power magnification of the papillary adenomyoepithelioma component of the primary tumor (scale bar, 1mm). **(q)** The adenomyoepithelioma component of the ipsilateral breast recurrence is histologically similar to the primary lesion (scale bar, 100µm). **(r)** The invasive ductal carcinoma of no special type component of the ipsilateral breast recurrence is composed of solid sheets of pleomorphic epithelial cells (scale bar, 10µm). **(s)** Low power magnification of the axillary lymph node metastasis (scale bar, 1mm). Note that the biphasic papillary architecture is retained in the axillary lymph node metastasis, akin to the histologic pattern observed in the primary adenomyoepithelioma. **(t)** Medium power magnification of the myoepithelial-enriched component of the lymph node metastasis (scale bar, 100µm). **(u)** Medium power magnification of the epithelial-enriched component of the lymph node metastasis (scale bar, 100µm). **v-x AM46 (v)** Low power magnification showing the adenomyoepithelioma component (*, low right corner) and spindle cell metaplastic carcinoma (**, top left corner, scale bar, 1mm). **(w)** Medium power magnification of the tubular adenomyoepithelioma component (scale bar, 200µm). **(x)** Medium power magnification of the spindle cell metaplastic carcinoma, with fibromatosis-like features (scale bar, 200µm).

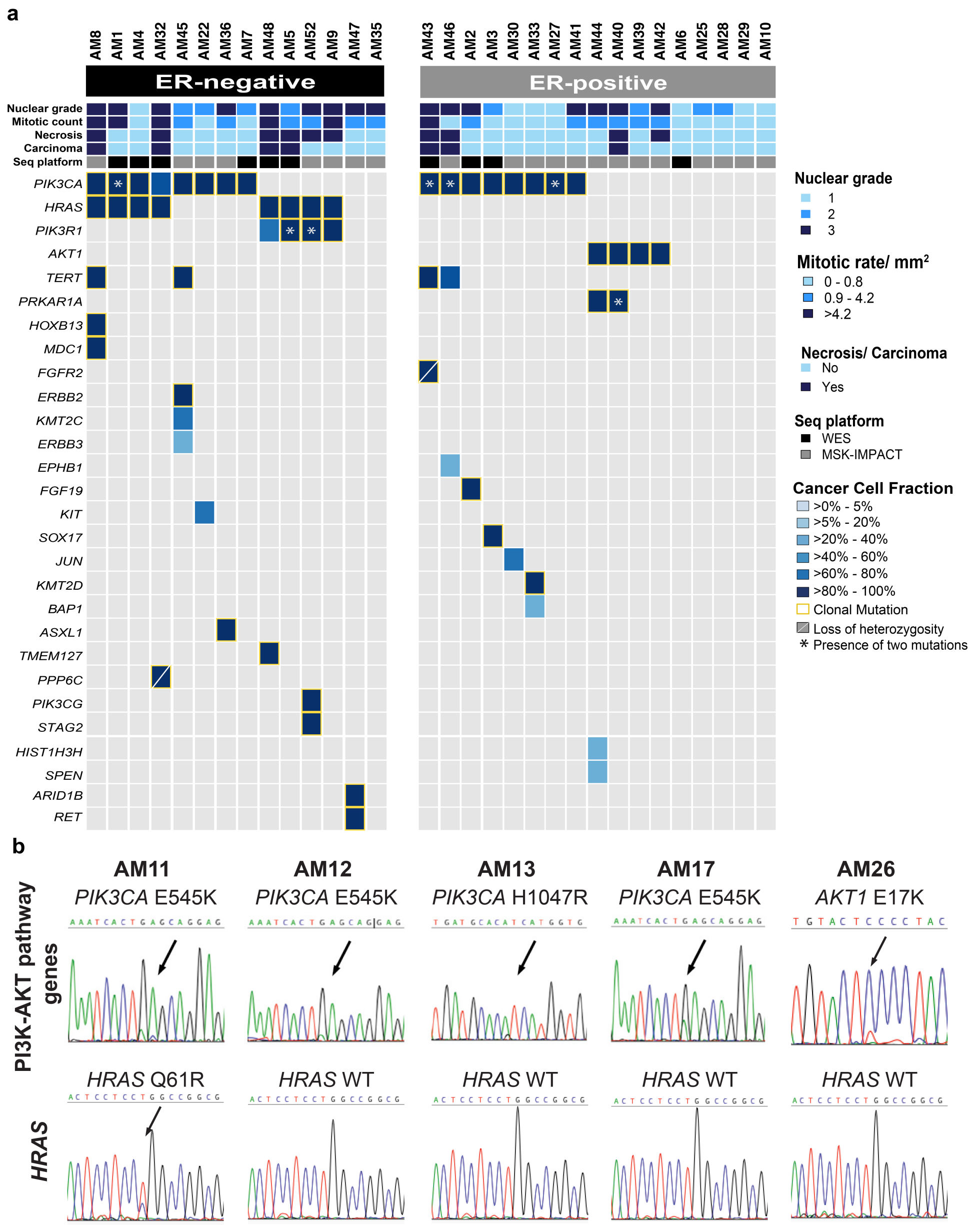

Supplementary Figure 2: Cancer cell fractions of non-synonymous somatic mutations identified in breast adenomyoepitheliomas by massively parallel sequencing, and Sanger sequencing traces of *HRAS*, *PIK3CA* and *AKT1* mutations identified in cases subjected only to Sanger sequencing.

(a) Heatmap depicting the cancer cell fractions of somatic mutations identified in the 31 breast adenomyoepitheliomas subjected to whole-exome (WES, n=10) or MSK-IMPACT (n=21) massively parallel sequencing, defined using ABSOLUTE. Cases are shown in columns (estrogen receptor (ER)-negative cases on the left; ER-positive cases on the right), and genes in rows. Histopathologic characteristics and information on the sequencing platforms are shown in the phenotype bar at the top. Mutations affecting the 410 cancer-related genes present in MSK-IMPACT are plotted, and their cancer cell fractions are color-coded according to the legend. Loss of heterozygosity (LOH) is represented by a diagonal bar. Clonal mutations are highlighted with orange boxes. The presence of two mutations in the same gene is indicated by an asterisk. Seq platform, sequencing platform employed. **(b)** Sanger sequence electropherograms of breast adenomyoepitheliomas analyzed by Sanger sequencing in which mutations affecting *HRAS*, *PIK3CA* or *AKT1* were detected.

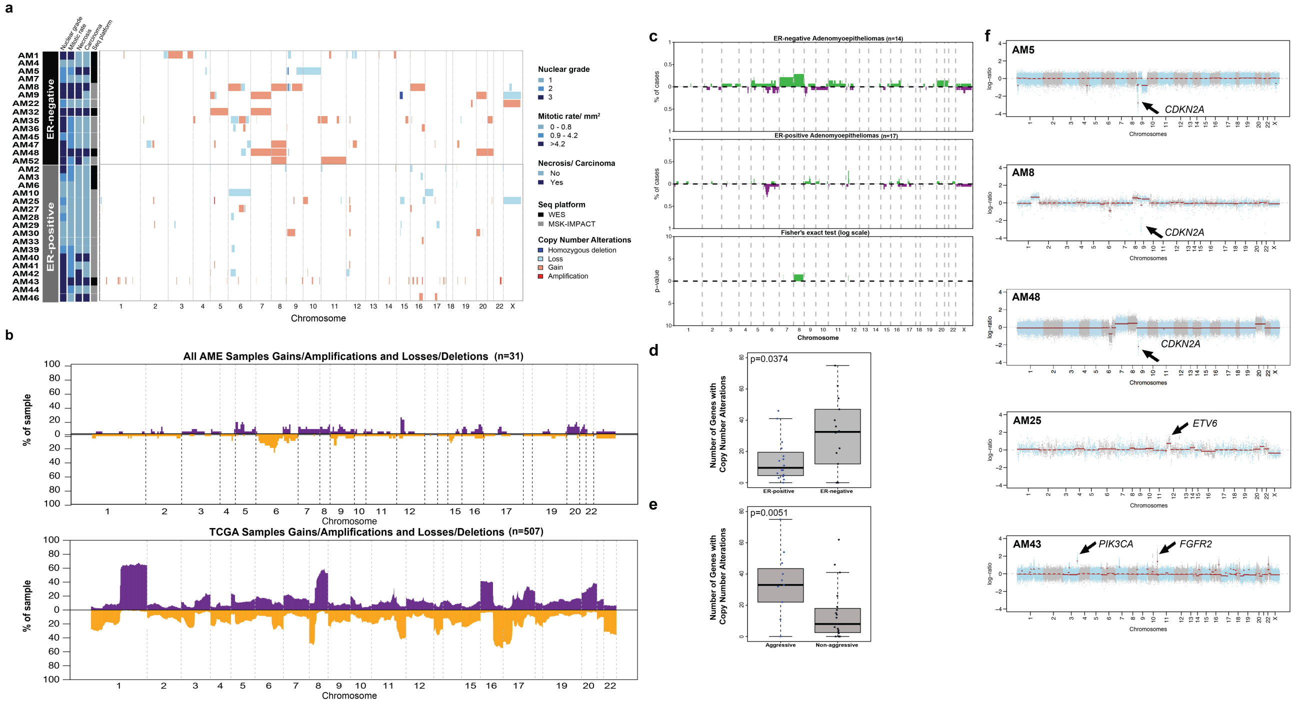

Supplementary Figure 3: Copy number alterations in breast adenomyoepitheliomas.

(a) Repertoire of copy number alterations present in the 31 adenomyoepitheliomas subjected to whole-exome (n=10) or MSK-IMPACT (n=21) massively parallel sequencing. Histopathologic features and the sequencing platforms employed for each sample are shown in the phenotype bar. Samples are represented in rows (estrogen receptor (ER)-negative cases on the top; ER-positive cases in the bottom) and the genomic position on the x-axis. Copy number alterations are depicted according to the color coding in the legend. **(b)** Frequency of copy number alterations in the 31 adenomyoepitheliomas (AMEs) analyzed with either whole-exome or MSK-IMPACT massively parallel sequencing, and in 507 breast cancers from The Cancer Genome Atlas (TCGA) study. Genomic positions are displayed along the x-axis and the percentage of samples with alterations (gains, purple; losses, yellow) at each location displayed on the y-axis. **(c)** Frequency plot and multi-Fisher's exact test comparison of copy number gains and losses in 14 ER-negative and 17 ER-positive breast adenomyoepitheliomas analyzed with whole-exome (n=10) or MSK-IMPACT (n=21) massively parallel sequencing. The frequency of gains (green) or losses (purple) for each gene is plotted on the y-axis, according to their genomic position on the x-axis. Inverse Log_{10} values of the Fisher's exact test p-values are plotted according to genomic location (x-axis). **(d)** Boxplot of the number of genes affected by copy number alterations in 14 ER-negative and 17 ER-positive breast adenomyoepitheliomas. Comparison was performed using Wilcoxon test. **(e)** Boxplot of the number of genes affected by copy number alterations in adenomyoepitheliomas displaying necrosis and/ or high mitotic rates ("Aggressive", n=11), compared to those without ("Not aggressive", n=20). Comparison was performed using Wilcoxon test. **(f)** Copy number profiles of AM5, AM8 and AM48, which harbored *CDKN2A* homozygous deletions, and of AM25 and AM43, which harbored amplifications of selected oncogenes. The chromosome location is plotted on the x-axis and the Log_2 ratios on the y-axis. Arrows highlight homozygous deletions or amplifications.

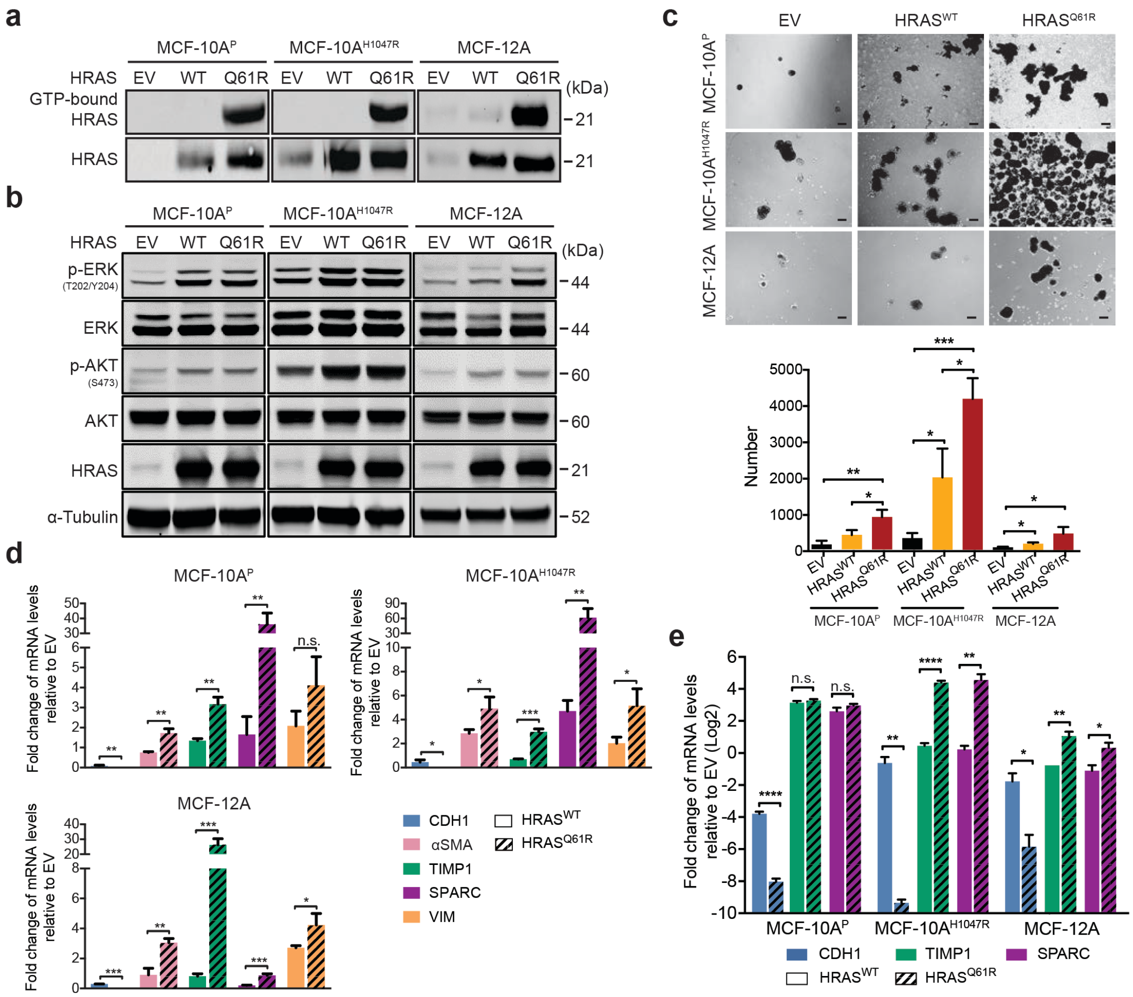

Supplementary Figure 4: Impact of mutant HRAS^{Q61R} expression on MAPK and AKT signaling pathways, mammosphere formation and expression of myoepithelial markers (qRT-PCR analysis) in non-malignant breast epithelial cells.

(a) Enrichment and pull-down of the active form of HRAS (GTP-HRAS) from endogenous lysates of MCF-10A^{P}, MCF-10A^{H1047R} and MCF-12A cells stably expressing empty vector (EV), HRAS^{WT} or mutant HRAS^{Q61R} protein, as assessed by western blot analysis using an anti-HRAS specific antibody. **(b)** Representative western blot analysis of HRAS, total and phosphorylated protein levels of ERK and AKT in MCF-10A^{P}, MCF-10A^{H1047R} and MCF-12A cells stably expressing EV, HRAS^{WT} or HRAS^{Q61R}. α-Tubulin was used as a loading control. **(c)** Representative micrographs of tumor spheres from MCF-10A^{P}, MCF-10A^{H1047R} and MCF-12A cells stably expressing EV, HRAS^{WT} or mutant HRAS^{Q61R} formed for 8 days (top; scale bars, 400μm). Quantitation of the number of mammospheres was performed by Fiji (Image J software). **(d)** Quantitative assessment of transcripts of *ACTA2* (αSMA), *TIMP1*, *CDH1*, *SPARC* and *VIM* in MCF-10A^{P}, MCF-10A^{H1047R}, and MCF-12A cells stably expressing HRAS^{WT} or mutant HRAS^{Q61R} cultured in monolayer. **(e)** Quantitative assessment of transcripts of *CDH1, SPARC* and *TIMP1* in MCF-10A^{P}, MCF-10A^{H1047R}, and MCF-12A cells stably expressing HRAS^{WT} or mutant HRAS^{Q61R} cultured in three-dimensional basement membrane for 10 days. In **(d)** and **(e)**, the expression levels were normalized to *GAPDH* expression, and comparisons of mRNA expression levels were performed between HRAS^{WT} and HRAS^{Q61R}, both relative to EV. **(c-e)** Data are representative of three independent experiments. Error bars, s.d. of mean (n=3). *$P < 0.05$, ** $P < 0.01$, *** $P < 0.001$, **** $P < 0.0001$; two tailed unpaired *t*-tests.

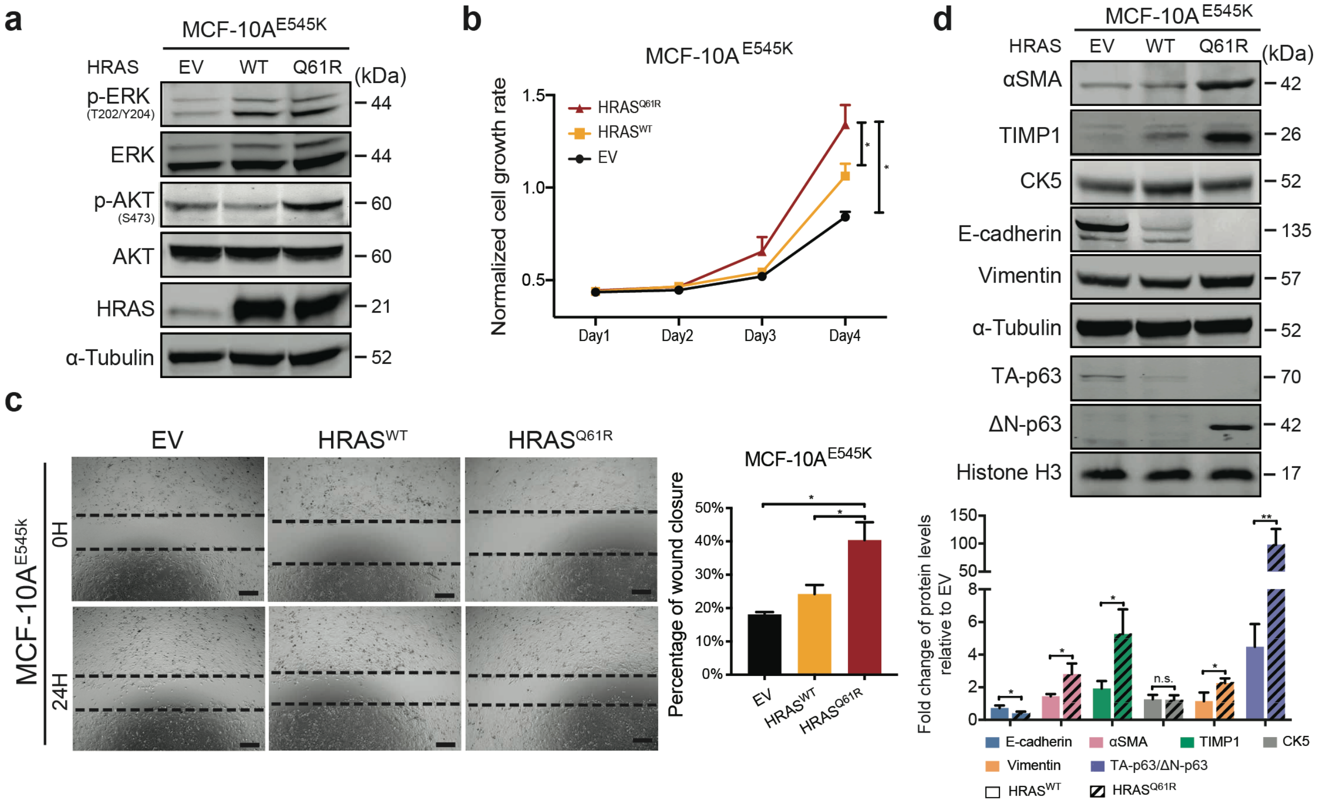

Supplementary Figure 5: Mutant HRAS^{Q61R} expression results in the acquisition of oncogenic properties and a partial myoepithelial phenotype in non-malignant breast epithelial cells with mutant *PIK3CA*^{E545K}.

(a) Representative western blot of duplicate analysis of HRAS, total and phosphorylated protein levels of ERK and AKT in MCF-10A^{E545K} cells transiently transfected with empty vector (EV), HRAS^{WT} or HRAS^{Q61R}. α-Tubulin was used as a loading control. **(b)** MTT cell proliferation assay of MCF-10A^{E545K} cells transfected with EV (black), HRAS^{WT} (yellow) or mutant HRAS^{Q61R} (red). **(c)** The migratory effects of MCF-10A^{E545K} transiently transfected with EV, HRAS^{WT} or mutant HRAS^{Q61R} were analyzed using the wound healing assay at 0 and 24 hours. Scale bars, 500µm. In **(b)** and **(c)**, data are representative of three independent experiments. Error bars, s.d. of mean (n=3). *$P < 0.05$; two tailed unpaired *t*-tests. **(d)** Representative western blot (top) of a triplicate analysis of total protein expression of alpha-smooth muscle actin (αSMA), TIMP1, cytokeratin 5 (CK5), E-cadherin, and vimentin, and nuclear protein expression of ΔN-p63 and TA-p63 in MCF-10A^{E545K} cells transiently transfected with EV, HRAS^{WT} or mutant HRAS^{Q61R}. α-Tubulin and Histone H3 were used as protein loading controls for total and nuclear protein expression, respectively. Quantification (bottom) using LI-COR is shown based on experiments done in triplicate. Comparisons of protein levels were performed between HRAS^{WT} and mutant HRAS^{Q61R}, both relative to EV. Error bars, s.d. of mean (n=3). n.s. = not significant, *$P < 0.05$, **$P < 0.01$; two tailed unpaired *t*-test.

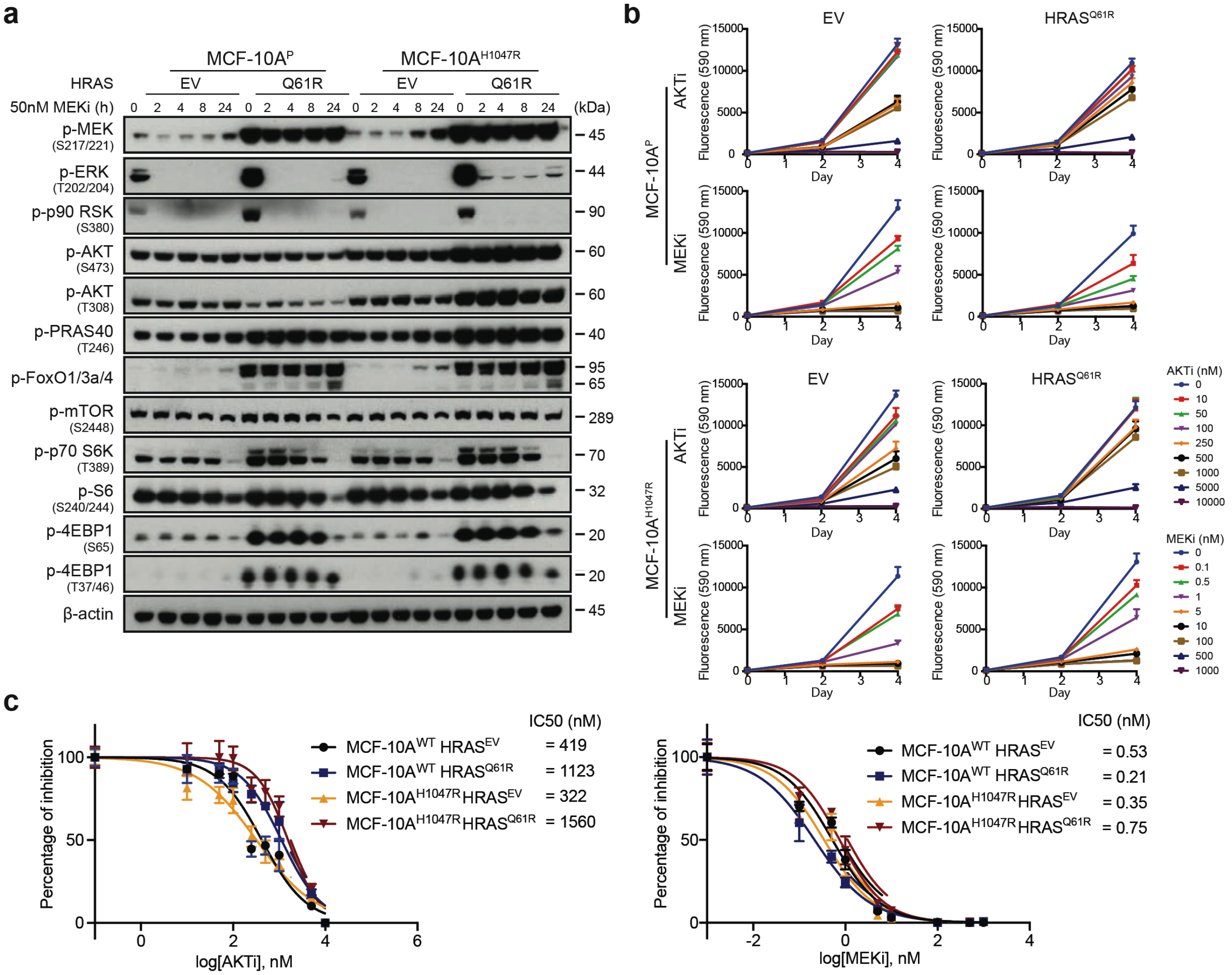

Supplementary Figure 6: Impact of AKT and MEK inhibition on PI3K-AKT and MAPK signaling pathways and proliferation in non-malignant breast epithelial cells expressing mutant HRAS^{Q61R}. **(a)** Representative western blot analysis of p-MEK (S217/221), p-ERK1/2 (T202/Y204), p-p90 RSK (S380), p-AKT (S473), p-AKT (T308), p-PRAS40 (T246), p-FOXO1/3a/4, p-mTOR (S2448), p-p70 S6K (T389), p-S6 (S240/244), p-4EBP1 (S65) and p-4EBP1 (T37/46) protein in MCF-10A^{P} and MCF-10A^{H1047R} cells stably expressing empty vector (EV) or mutant HRAS^{Q61R} treated with 50nM MEK inhibitor (MEKi, GSK212) at different time points. β-actin was used as a protein loading control. **(b)** Dose-dependent inhibitory effects in MCF-10A^{P} and MCF-10A^{H1047R} cells expressing EV or mutant HRAS^{Q61R} treated with AKT inhibitor (AKTi, MK2206) or MEKi for 2 and 4 days. Data are representative of three independent experiments. Error bars, s.d. of mean (n=3). **(c)** Median inhibitory concentration (IC50) of AKTi and MEKi in MCF-10A^{P} and MCF-10A^{H1047R} cells expressing EV or mutant HRAS^{Q61R} treated for 4 days. Triplicate experiments were repeated at least twice with similar results.

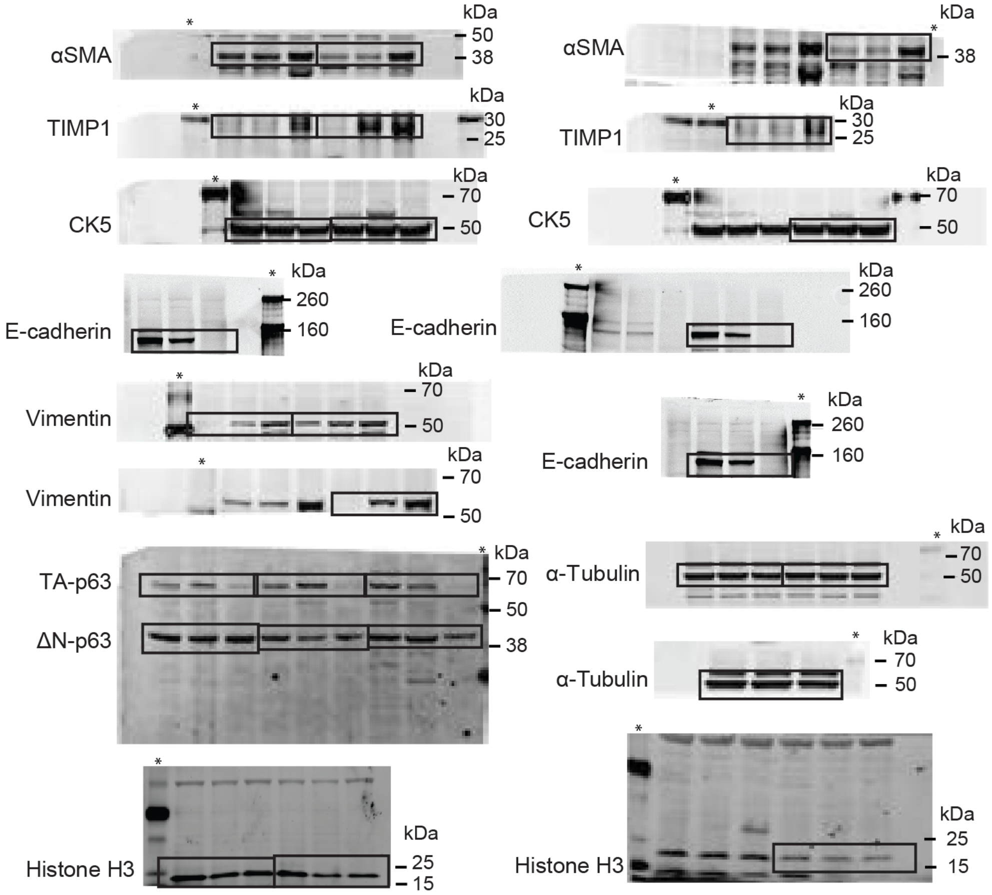

Supplementary Figure 7: Unprocessed images of western blots.
Unprocessed images of scanned immunoblots shown in Figure 6 of the main manuscript are provided. The asterisks indicate the ladder.

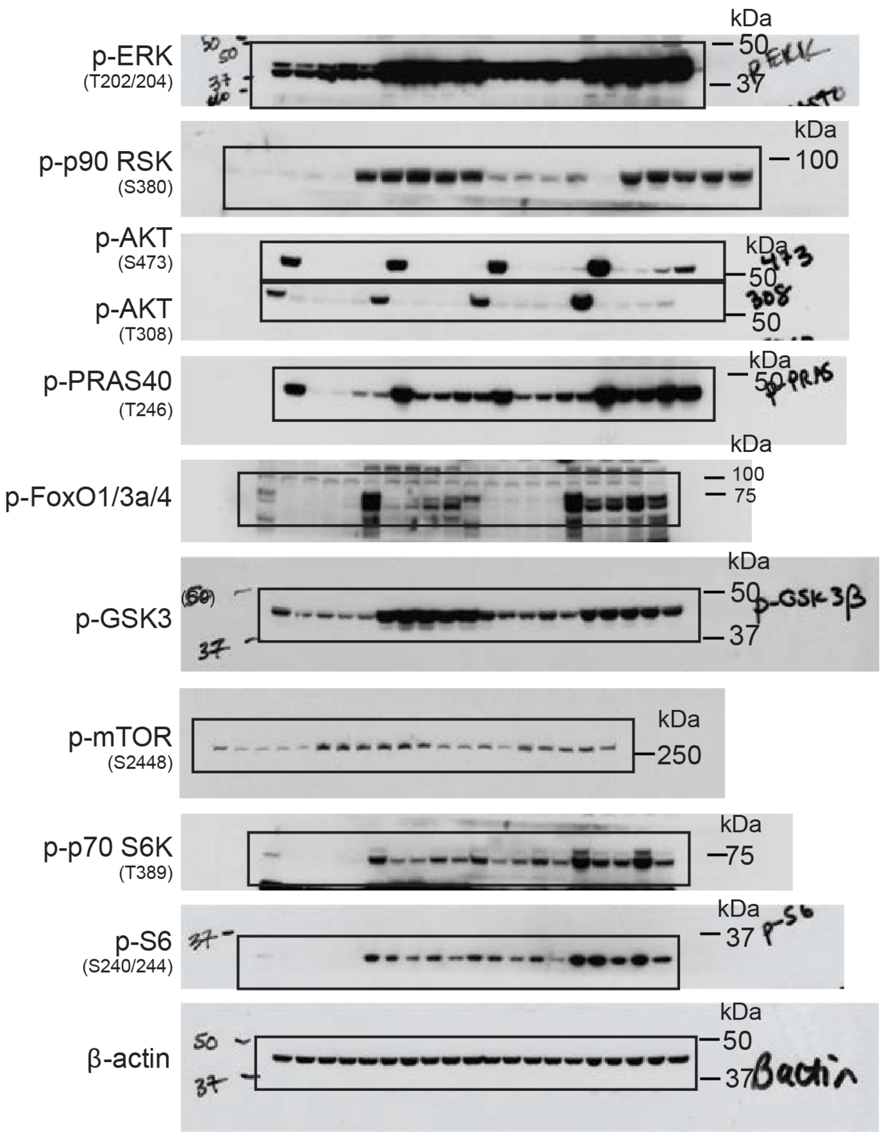

Supplementary Figure 8: Unprocessed images of western blots.
Unprocessed images of scanned immunoblots shown in Figure 7 of the main manuscript are provided.

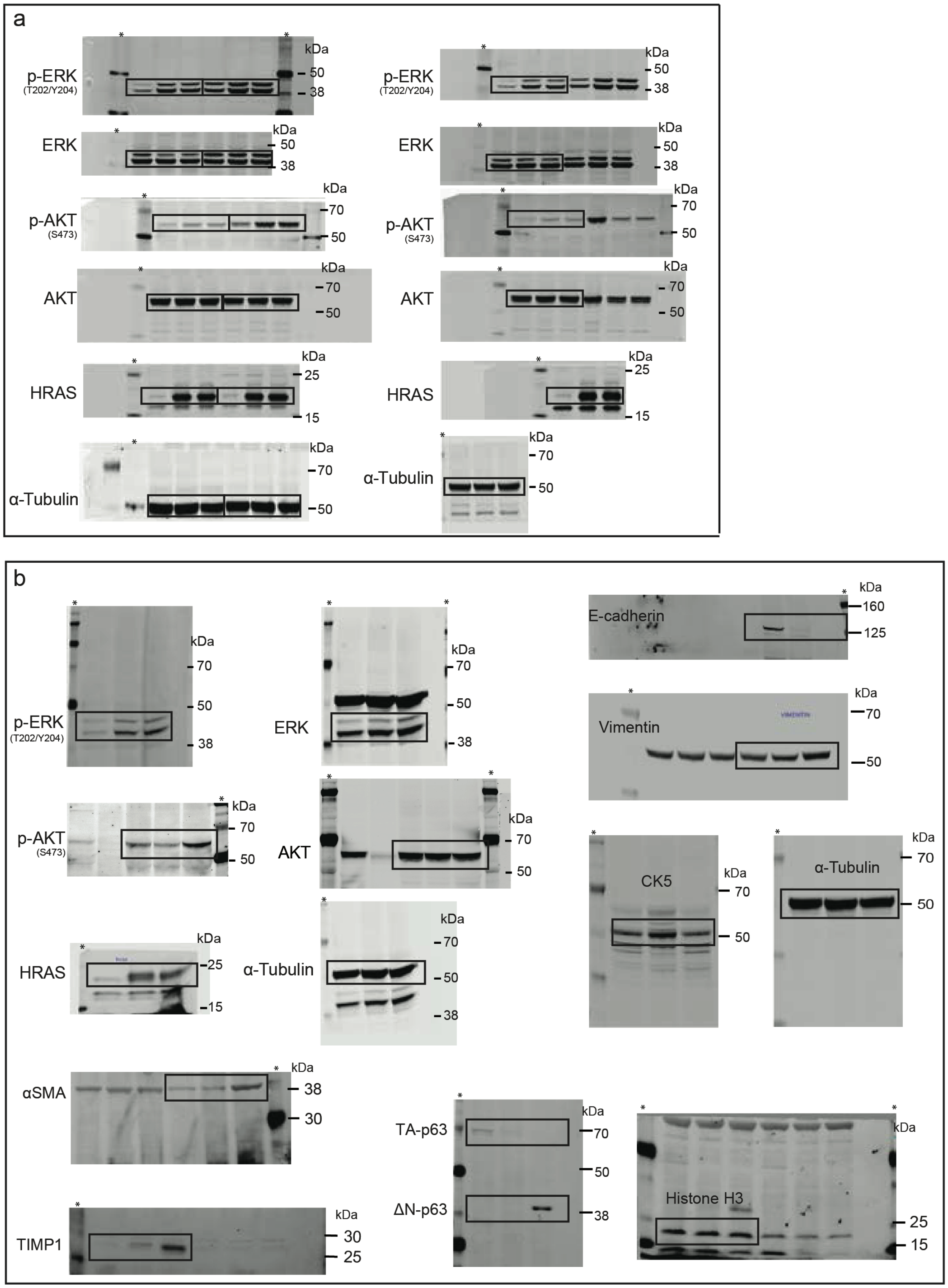

Supplementary Figure 9: Unprocessed images of western blots.
Unprocessed images of scanned immunoblots shown in **(a)** Supplementary Figure 4 and **(b)** Supplementary Figures 5 are provided. The asterisks indicate the ladder.

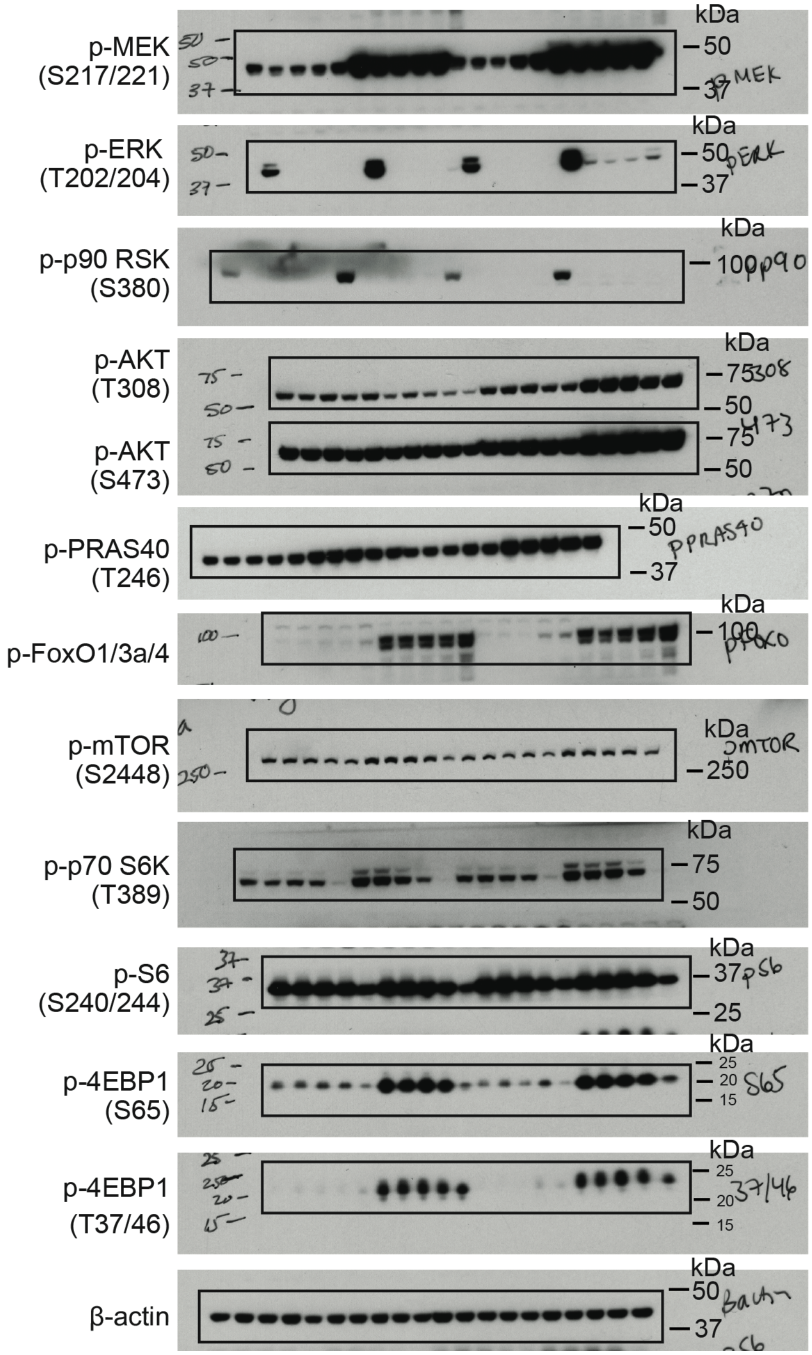

Supplementary Figure 10: Unprocessed images of western blots.
Unprocessed images of scanned immunoblots shown in Supplementary Figure 6 are provided.
