# Supplementary material for: Recurrent hotspot mutations in HRAS Q61 and PI3K-AKT pathway genes as drivers of breast adenomyoepitheliomas
Source: Nat Commun. 2018 May 8;9:1816. doi: 10.1038/s41467-018-04128-5 (PMC5940840; doi:10.1038/s41467-018-04128-5)
Supplement: Supplementary file 2 — Description of Additional Supplementary Files [file 41467_2018_4128_MOESM2_ESM.pdf]

Description of Additional Supplementary Files

Supplementary Data 1. Clinicopathologic and immunohistochemical features, and *HRAS, PIK3CA, AKT1* and *PIK3R1* mutation status of 43 breast adenomyoepitheliomas included in this study.

Supplementary Data 2: Sequencing statistics, number of somatic mutations identified and validation rate of mutations in breast adenomyoepitheliomas subjected to whole-exome sequencing or MSK-IMPACT targeted sequencing.

Supplementary Data 3: List of somatic mutations identified in breast adenomyoepitheliomas and associated lesions by whole-exome sequencing or MSK-IMPACT massively parallel sequencing.

Supplementary Data 4: Comparison of the number of somatic mutations affecting *HRAS, PIK3CA* and *AKT1* between the breast adenomyoepitheliomas analyzed in this study and common forms of invasive breast cancer from The Cancer Genome Atlas (TCGA) and International Cancer Genome Consortium (ICGC) studies.
